# Single-Cell RNA Transcriptomics and Multi-omics Analyses Reveal the Clinical Effects of Acupuncture on Methadone Reduction

**DOI:** 10.34133/research.0741

**Published:** 2025-06-24

**Authors:** Yiming Chen, Baochao Fan, Jingchun Zeng, Yutian Zou, Chenyang Tao, Chen Chen, Peiming Zhang, Jian Liang, Fangfang Qi, Hailin Tang, Liming Lu

**Affiliations:** ^1^ Clinical Research and Big Data Laboratory, South China Research Center for Acupuncture and Moxibustion, Medical College of Acu-Moxi and Rehabilitation, Guangzhou University of Chinese Medicine, Guangzhou, China.; ^2^ Department of Acupuncture, First Affiliated Hospital of Guangzhou University of Chinese Medicine, Guangzhou, China.; ^3^ State Key Laboratory of Oncology in South China, Guangdong Provincial Clinical Research Center for Cancer, Sun Yat-Sen University Cancer Center, Guangzhou, China.; ^4^ Dana-Farber Cancer Institute, Harvard Medical School, Boston, MA 02215, USA.; ^5^ Jiangsu Medical College, Yancheng, China.; ^6^ The Eighth Clinical College of Medicine, Guangzhou University of Chinese Medicine, Foshan, Guangdong, China.; ^7^ School of Pharmaceutical Sciences, State Key Laboratory of Traditional Chinese Medicine Syndrome, Guangzhou University of Chinese Medicine, Guangzhou, China.; ^8^ Department of Neurology, Mayo Clinic, Rochester, MN 55905, USA.

## Abstract

Opioid use disorders (OUDs) pose a substantial global health burden, with methadone maintenance treatment (MMT) widely adopted as an intervention; however, MMT is associated with immunosuppression, metabolic disturbances, and dysbiosis of the gut microbiota. Despite the potential of acupuncture in reducing methadone dosages and opioid addiction, the underlying biological mechanisms remain unclear. Therefore, we aimed to integrate clinical trial data with multi-omics analysis, including single-cell sequencing, transcriptomics, metabolomics, and metagenomics, to evaluate the effects of acupuncture in patients undergoing MMT. We collected peripheral blood mononuclear cells, plasma, and fecal samples from 48 MMT participants in a randomized, placebo-controlled trial. Participants were divided into acupuncture (*n* = 25) and sham-acupuncture (*n* = 23) groups. After 8 weeks of intervention, 84% of patients in the acupuncture group achieved ≥20% reduction in methadone dosage, compared to 39% in the sham-acupuncture group (*P* < 0.01). Our findings revealed that acupuncture may activate the defense response to viruses, with altered immune cell functions in classical monocytes correlating with clinical responses to reduced methadone doses. Acupuncture might ameliorate intestinal microbial disruptions caused by OUD by up-regulating *Bilophila* and modulating bile acid metabolism. Furthermore, acupuncture up-regulated galectin-9 (LGALS9)-mediated intercellular communication between classical monocytes and other immune subsets. To further validate the mechanistic link between bile acid metabolism and immune regulation, we conducted in vitro experiments using THP-1 monocytes treated with cholic acid. The results showed that bile acid exposure suppressed galectin-9 and IFN-γ expression, while low-dose bile acid (simulating acupuncture effects) partially reversed this effect. These findings support a bile acid–galectin–interferon axis that may be modulated by acupuncture in OUD. Collectively, our results provide clinical and mechanistic evidence supporting acupuncture as a potential adjunct therapy to mitigate the adverse effects of long-term opioid use.

## Introduction

The 2024 World Drug Report indicated that an estimated 60 million individuals suffer from opioid use disorders (OUDs), presenting a substantial global health challenge [1]. OUDs are major contributors to the development of severe diseases and drug-related fatalities worldwide, including overdoses, infectious diseases, liver diseases, cardiovascular diseases, and mental health issues [2]. The 2021 China Drug Situation Report states that approximately 1.4 million people use illicit drugs, with opioids and methamphetamines accounting for 90.5% of usage [3]. In response to this public health issue, methadone maintenance treatment (MMT) has been broadly adopted as an effective intervention to reduce opioid use [4].

MMT has shown positive outcomes in reducing illicit drug use [5,6]; however, numerous clinical and fundamental studies have confirmed that opioid abuse leads to various side effects, including addiction, dysbiosis of the gut microbiota, disruption in metabolism leading to persistent neurochemical disturbance, and immunosuppression [7–10]. Individuals with substance use disorders or those on chronic opioid therapy face increased risks for bacterial and viral infections compared with the health status of the general population [11]. Opioid exposure leads to the suppression of the antiviral gene program in naive monocytes [12]. Chronic opioid exposure leads to a depletion of short-chain fatty acids and short-chain-fatty-acid-producing bacteria, thereby leading to increased gastrointestinal tract permeability. This increased permeability facilitates the translocation of microbes and endotoxins from the gut lumen, subsequently triggering a systemic immune response [13]. The disruption of metabolites and increased gut epithelium permeability are likely pivotal in driving the addiction cycle via impacts on the immune system [14].

Acupuncture is a therapeutic technique that involves inserting fine metal needles into specific acupoints, followed by manual or electrical manipulation to elicit physiological responses [15]. The theoretical foundation of acupuncture lies in its ability to balance and maintain the immune system and intestinal microbiota, aiming to regulate yin and yang, support bodily vitality, and eliminate pathogenic factors [16–20]. Consequently, the World Health Organization has acknowledged acupuncture as a suitable nonpharmacological treatment for substance dependence [21]. Concordantly, our previous clinical study confirmed that acupuncture could reduce methadone dosage and alleviate opioid cravings among individuals receiving MMT [22–24]. However, prior studies have typically focused on isolated biological domains and that the combined, systems-level mechanisms underlying acupuncture’s clinical effects in MMT remain poorly understood, limiting the broader adoption of acupuncture in practice [15].

As a potential tool for understanding the molecular mechanisms of medical interventions, omics technology is increasingly utilized in clinical research [25]; however, conventional bioinformatics analysis methods often confine themselves to pairwise comparisons in highly heterogeneous case–control studies [26]. Currently, omics analyses of randomized controlled trials typically focus on examining changes pre- and post-interventions [27,28]. These methods seldom account for time-related effects and uneven baselines in intergroup comparisons of placebo-controlled trials, which pose challenges in identifying complex heterogeneities resulting from acupuncture interventions. Integrating data from different physiological spaces (gut, blood, brain, and tissues) can offer a holistic view of acupuncture’s overall effects [29].

Therefore, we performed a multi-omics analysis of randomized placebo-controlled trials to assess the molecular characteristics of acupuncture in patients undergoing MMT using various methods, including single-cell RNA sequencing (scRNA-seq), bulk RNA sequencing (bulk RNA-seq), whole-metagenome shotgun sequencing (WMS), broad-spectrum metabolomics analysis via liquid chromatography–mass spectrometry (LC–MS), and gas chromatography–mass spectrometry (GC–MS) in our study. We aimed to provide scientific evidence of the effectiveness of acupuncture in mitigating methadone-induced immunosuppression and alleviating overall metabolic disturbances.

## Results

### Patient characteristics

Between April 2022 and April 2023, 118 of the 142 screened patients were enrolled at baseline and randomly assigned to either the acupuncture or the sham-acupuncture group. A total of 48 participants provided paired biological samples for at least one specimen type (peripheral blood mononuclear cells [PBMCs], stool, or plasma), which were subsequently used for omics analyses. The demographic and clinical characteristics of the patients are presented in the Table with no statistically significant differences observed between the acupuncture and sham-acupuncture groups (*P* > 0.05).

**Table.** Patient characteristics

Characteristic	Acupuncture group (*n* = 25)	Sham-acupuncture group (*n* = 23)	*t*/*U*/χ^2^	*P* value
Methadone dose, median (IQR), mg	50.00 (35.00)	45.00 (35.00)	290.00	0.959
Age, mean (SD)	48.08 (7.54)	49.43 (8.02)	−1.35	0.183
Sex, no. (%)				
Male	22 (88.00)	23 (100.00)		
Female	3 (12.00)	0 (0.00)		
BMI, median (IQR), kg/m^2^	21.03 (5.06)	23.39 (4.86)	374.00	0.074
Marital status (%)			0.65	0.724
Married	17 (68.00)	18 (78.30)		
Single	5 (20.00)	3 (13.0)		
Divorced	3 (12.00)	2 (8.70)		
Occupation (%)			0.64	0.424
Employed	17 (68.00)	18 (78.30)		
Unemployed	8 (32.00)	5 (21.70)		
Education (%)			2.44	0.119
Primary or middle school	17 (68.00)	20 (87.00)		
High school or university	8 (32.00)	3 (13.00)		
Years of opioid use, mean (SD), y	13.16 (5.54)	14.87 (5.33)	1.00	0.321
Route of previous opioid use, no. (%)			ND	0.140
Injection	18 (72.00)	21 (91.30)		
Nasal or oral	7 (28.00)	2 (8.70)		

IQR, interquartile range; SD, standard deviation; BMI, body mass index; ND, no data

### Clinical outcomes

By the end of the intervention (week 8), 21 of 25 patients (84.00%) undergoing MMT in the acupuncture group achieved a methadone reduction of ≥ 20%, compared with 9 of 23 patients (39.13%) in the sham-acupuncture group (χ^2^ = 10.29, *P* = 0.001). No adverse events were reported in either group throughout the study period.

### Acupuncture was associated with defense response to viruses

**Fig. 1.**
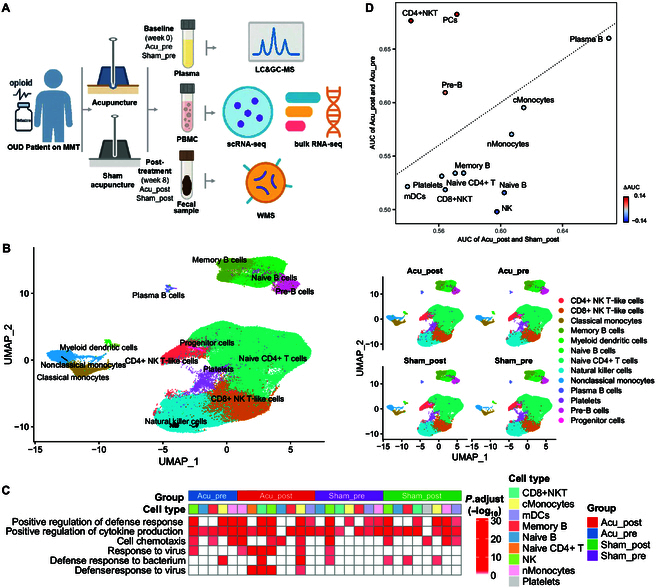
Single-cell landscape of peripheral blood mononuclear cells (PBMCs) in the acupuncture and sham-acupuncture groups, pre- and post-intervention. (A) Trial design. (B) Uniform manifold approximation and projection (UMAP) visualization of all identified cell clusters. The left panel represents cell types by colors, and the right panel is divided according to the sample origin. (C) Gene Ontology (GO) term enrichment analysis of the top 100 marker genes. (D) The scatter plot of area under the curve (AUC) reflects the degrees of differences in cell types between the Acu_post vs. Acu_pre and Acu_post vs. Sham_post groups. OUD, opioid use disorder; MMT, methadone maintenance treatment; scRNA-seq, single-cell RNA sequencing; bulk RNA-seq, bulk RNA sequencing; GC–MS, gas chromatography–mass spectrometry; LC–MS, liquid chromatography–mass spectrometry; WMS, whole-metagenome shotgun sequencing; NK, natural killer; CD8+NKT, CD8+ NK T-like cells; CD4+NKT, CD4+ NK T-like cells; cMonocytes, classical monocytes; mDCs, myeloid dendritic cells; nMonocytes, nonclassical monocytes.

We collected 7 paired samples of PBMCs from 4 patients in the acupuncture group and 3 in the sham-acupuncture group before and after the intervention for scRNA-seq analysis. Following quality control, we analyzed data from 128,751 single cells. Based on the cellular expression of prototypical signature genes, we annotated 13 cell clusters, including naive CD4+ T cells, natural killer (NK) cells, CD4+ NK T-like (CD4+NKT) cells, CD8+ NK T-like (CD8+NKT) cells, pre-B cells, naive B cells, memory B cells, plasma B cells, classical monocytes (cMonocytes), nonclassical monocytes (nMonocytes), myeloid dendritic cells (mDCs), progenitor cells (PCs), and platelets. All 13 clusters were present in PBMC samples from the pre-acupuncture (Acu_pre), post-acupuncture (Acu_post), pre-sham (Sham_pre), and post-sham (Sham_post) intervention groups (Fig. 1B).

We further compared gene expression levels within the different cell clusters to assess intragroup differences before and after the acupuncture intervention, as well as intergroup differences between acupuncture and sham-acupuncture interventions (Fig. S1). Interferon-stimulated genes (ISGs), such as *ISG15*, *IFIT1*, *IFIT3*, *IFITM2*, and *IFITM3*, were up-regulated in NK cells, CD8+NKT cells, cMonocytes, nMonocytes, and plasma B cells.

We then identified marker genes in each group and cell cluster for subsequent Kyoto Encyclopedia of Genes and Genomes (KEGG) pathway (top 1,000 marker genes) and Gene Ontology (GO) term (top 100 marker genes) enrichment analyses. The genes identified in the Acu_post group, particularly in cMonocytes, were enriched for biological processes associated with the GO terms “response to virus”, “defense response to virus”, and “defense response to bacterium” (Fig. 1C and Fig. S2).

Our Augur models indicated that the cell clusters most effective at distinguishing the Acu_post samples from the Acu_pre samples included PCs, CD4+NKT cells, plasma B cells, pre-B cells, and cMonocytes. In contrast, the cell clusters most effective at distinguishing the Acu_post samples from the Sham_post samples included plasma B cells, cMonocytes, nMonocytes, naive B cells, and NK cells (Fig. S3). The scatter plot shown in Fig. 1D illustrates that cMonocytes and plasma B cells shared immune cell populations during acupuncture therapy, based on the analysis of both models. Consequently, a higher perturbation of plasma B cells and cMonocytes was induced by acupuncture when considering both time-related and placebo effects. Conversely, the alterations observed in CD4+NKT cells and PCs represented perturbations evolving over time, with minimal perturbations observed in mDCs.

These results suggest that acupuncture altered gene expression levels in various immune cell clusters, particularly in cMonocytes and plasma B cells, enhancing the defense response to virus and differentiating these effects from those observed in the sham-acupuncture group.

### Acupuncture amplified cellular interactions between cMonocytes and other immune cells via the galectin pathway

**Fig. 2.**
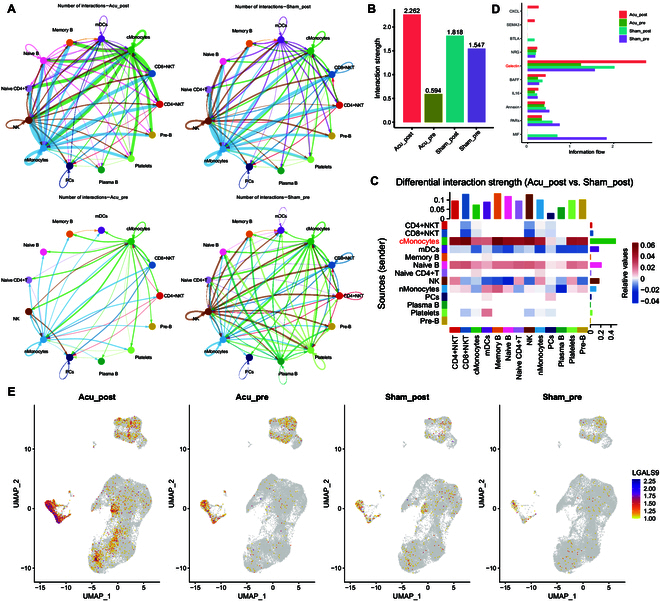
Cell–cell interaction network of PBMCs in the acupuncture and sham-acupuncture groups, pre- and post-intervention. (A) Circle plots showing the total interaction strengths (weights) between any 2 cell clusters with a different sample origin. (B) The degree of interaction in the inferred cell–cell communication networks across samples from different group. (C) Heatmap depicting the total interaction strengths (weights) between any 2 cell clusters from the Acu_post and Sham_post groups. The sum of the column values (incoming signaling) is represented by the top colored bar plot, and the sum of the row values (outgoing signaling) is shown by the right colored bar plot. Red (or blue) represents an increase (or decrease) in values. (D) The information flow and interaction strength of each signaling pathway in all groups. (E) DimPlot analysis showing LGALS9 expression.

We investigated the cell–cell interaction network based on the expression of various ligand–receptor pairs. Interaction strengths were greater in the Acu_post group compared with those in the control groups, including Acu_pre, Sham_pre, and Sham_post (Fig. 2A and B). We observed more interactions between cMonocytes and naive CD4+ T cells, NK cells, CD4+NKT cells, CD8+NKT cells, pre-B cells, naive B cells, memory B cells, nMonocytes, mDCs, and platelets in the Acu_post group, primarily via the galectin signaling pathway (LGALS9–CD44 or LGALS9–CD45 signaling) than in the Sham_post group (Fig. 2C and D and Fig. S4). *LGALS9* was expressed at higher levels in monocyte subpopulations after acupuncture (Fig. 2E). Collectively, these findings suggest that acupuncture influenced interactions between cell clusters, potentially affecting galectin expression, immune cell functions, and inflammation regulation.

### cMonocytes and platelets were correlated with clinical response to acupuncture during methadone reduction

**Fig. 3.**
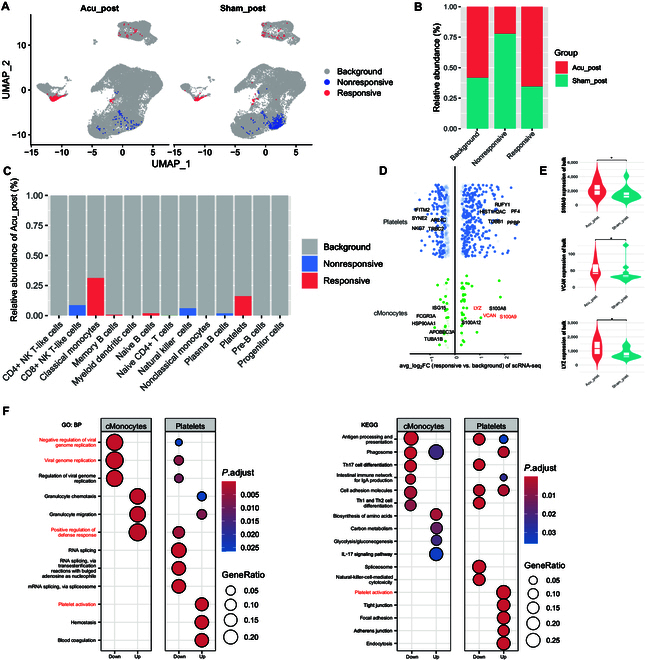
Identification of cell subpopulations associated with the bulk-sample phenotype. (A) UMAP visualization of responsive, nonresponsive, and background cells. (B) The relative abundances of responsive, nonresponsive, and background cells in the Acu_post and Sham_post groups. (C) The proportional fractions of the different identified cell types in the Acu_post group. (D) Volcano plot showing gene expression fold changes (FCs) (log_2_ scale) for each clinically relevant cell type compared with the corresponding values in background cells. (E) Violin plots showing gene expression levels of *S100A9*, *VCAN*, and *LYZ* in the Acu_post and Sham_post groups based on bulk RNA-seq data. *P < 0.05, as determined using the Wilcoxon test. (F) Enrichment analysis of differentially expressed genes (DEGs) in each clinically relevant cell type, based on GO (left panel) and Kyoto Encyclopedia of Genes and Genomes (KEGG) (right panel) analyses. BP, biological process; mRNA, messenger RNA.

To validate the scRNA-seq results, we performed bulk RNA-seq analysis on PBMCs from the same cohort, which included 20 patients in the acupuncture group and 15 in the sham-acupuncture group, both pre- and post-intervention. To assess the clinical roles of the cell clusters identified, we used Scissor to identify subclusters that correlated with the bulk-sample phenotype. cMonocytes and platelets correlated with methadone reduction response, whereas CD8+NKT cells did not show a response (Fig. 3A and C and Fig. S5). Generally, the number of PBMCs demonstrating a clinical response was higher in the acupuncture group than in the sham-acupuncture group, whereas the number of cells showing nonresponse was higher in the sham-acupuncture group (Fig. 3B).

We further compared gene expression differences between clinically relevant cells and background cells in cMonocytes and platelets (Fig. 3D). We identified 33 up-regulated and 18 down-regulated genes in responsive cMonocytes and 255 up-regulated and 117 down-regulated genes in platelets. *S100A9*, *VCAN*, and *LYZ* were up-regulated in responsive cMonocytes between the acupuncture and sham-acupuncture groups in bulk RNA-seq (Fig. 3E, *P* < 0.05). Analysis of the responding cMonocytes showed that acupuncture enhanced the GO terms “positive regulation of defense response” and “granulocyte chemotaxis” but suppressed “viral genome replication” (Fig. 3F, left panel). Additionally, the acupuncture-responsive cells enhanced the KEGG terms “platelet activation”, “adherens junctions”, and “endocytosis” (Fig. 3F, right panel), suggesting a noteworthy and potentially undiscovered function of platelets during acupuncture treatment.

These results indicated that acupuncture treatment was related to methadone reduction through gene expression changes in cMonocytes and platelets, highlighting their roles in the clinical response.

### Pseudotime analysis of cMonocyte differentiation after acupuncture intervention

**Fig. 4.**
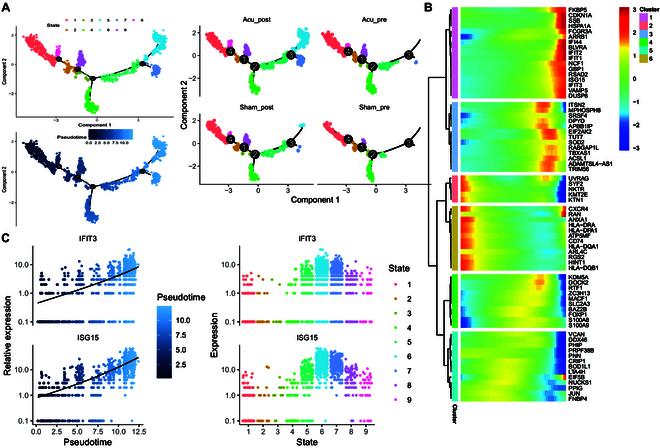
Pseudotime trajectory analysis of cMonocytes. (A) Overall pseudotime trajectories of cMonocytes in each group. The overall time-series trajectory begins on the left and ends on the right. (B) Pseudotime–expression clustering heatmap of genes in the Acu_post group. The horizontal axis corresponds to gene names, with pseudotime depicted on the vertical axis. (C) Expression of *IFIT3* and *ISG15* in the Acu_post group on branch clusters (left) and pseudotime trajectories (right).

We investigated the transformation of cMonocytes following acupuncture intervention using pseudotime analysis with Monocle2 to examine changes in their cell differentiation pathways. Four key differentiation nodes and 9 differentiation stages for cMonocytes were identified. The overall time-series trajectory begins on the left and ends on the right (Fig. 4A). The Acu_post group exhibited 2 specific differentiation nodes (nodes 6 and 7), whereas both the Acu_post and Sham_post groups shared one differentiation node (node 4). The distribution of cells across other differentiation nodes remained consistent (Fig. 4A). Temporal differential expression and *K*-means clustering analyses helped identify 6 temporally differentially expressed gene (DEG) sets (Fig. 4B). Gene cluster 1 showed high expression at the end of the temporal sequence, gene cluster 2 in the middle, and gene clusters 3 and 4 at the beginning. Gene clusters 5 and 6 exhibited low expression at both the beginning and the end. Gene cluster 1 contained several ISGs, including *IFI44*, *IFIT2*, *IFIT1*, *ISG15*, *IFIT3*, *GBP1*, and *RSAD2*. Within this cluster, *IFIT3* and *ISG15* were highly expressed at differentiation nodes 6 and 7 and at the end of the temporal sequence (Fig. 4C).

### Identification of acupuncture-specific gene sets from bulk RNA-seq data

**Fig. 5.**
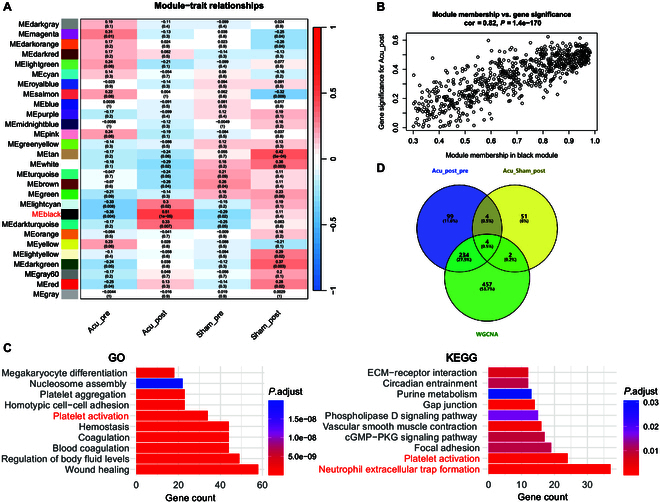
Identification of acupuncture-specific gene sets based on weighted gene coexpression network analysis (WGCNA) of bulk RNA-seq data. (A) Heatmap showing the relationship between coexpression modules and sample origins. (B) Pearson correlation analysis between the black module and Acu_post group. (C) GO terms (left panel) and KEGG (right panel) enrichment analysis of gene sets from the black module. (D) Venn diagram of the intersection between the acupuncture-specific gene set and DEGs, based on WGCNA (Acu_post vs. Acu_pre and Acu_post vs. Sham_post).

We conducted bulk RNA-seq, including top 15,000 genes for weighted gene coexpression network analysis (WGCNA) to identify coexpression modules. Notably, the black module demonstrated the highest correlation with the Acu_post group (*r* = 0.51, *P* < 0.01; Fig. 5A). Scatter plots indicated a positive correlation between the black module and the overall variation trend of the genes in the Acu_post group (correlation coefficient = 0.82, *P* < 0.01; Fig. 5B). The black module comprised 697 genes. GO term analysis showed that acupuncture was associated with platelet activation, including the regulation of coagulation and megakaryocyte differentiation (Fig. 5C). Additionally, KEGG pathway analysis indicated that acupuncture influenced neutrophil extracellular trap formation and platelet activation.

Using conventional differential analysis, we identified 410 DEGs between the Acu_post and Acu_pre groups and 73 DEGs between the Acu_post and Sham_post groups, with 8 DEGs common to both comparisons (Fig. 5D, Fig. S6, and Tables S1 and S2). The acupuncture-specific gene sets obtained from WGCNA included 240 of the aforementioned DEGs.

### Metabolomic profiling revealed acupuncture-induced changes in bile acid metabolism

We analyzed samples from 23 patients in the acupuncture group and 21 in the sham-acupuncture group before and after the intervention using LC–MS- and GC–MS-based untargeted plasma-metabolome profiling. The liquid- and gas-phase platforms helped identify 4,596 and 249 metabolites, respectively. Using conventional differential analysis, we identified 100 and 121 differential metabolites in the comparisons of Acu_post vs. Acu_pre and Acu_post vs. Sham_post, respectively, based on LC–MS data, with 15 overlapping metabolites between the 2 comparisons (Tables S5 and S6, *P* < 0.05). Similarly, GC–MS analysis revealed 48 and 61 differential metabolites for the same comparisons, with 22 metabolites shared between them (Tables S7 and S8, *P* < 0.05).

**Fig. 6.**
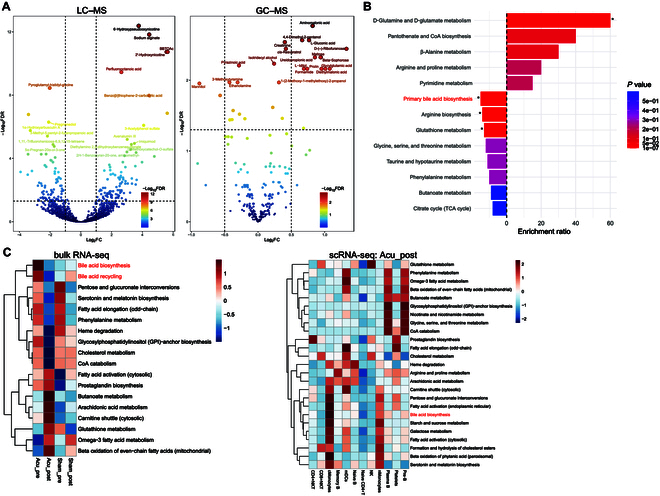
Metabolomics profiles related to the effect of acupuncture on methadone reduction. (A) Volcano plots showing longitudinally differential metabolites detected by LC–MS (left panel) or GC–MS (right panel) analysis. (B) KEGG pathway-enrichment analysis of longitudinally differential metabolites. Rightward movement represents up-regulation, and leftward movement represents down-regulation. *P < 0.05. (C) Flux-balance analysis of metabolic pathways observed using bulk RNA-seq data (left panel) or scRNA-seq data (right panel). FDR, false discovery rate.

Linear models with covariate adjustments (time and subjects) based on limma were used to identify variables associated with distinct metabolic outcomes resulting from acupuncture. We identified 197 liquid-phase and 29 gas-phase metabolites as potential unique biomarkers (Fig. 6A and Tables S3 and S4, adj. *P* < 0.05). Acupuncture increased the production of several metabolites, including 6-hydroxypseudooxynicotine, 2′-hydroxynicotine, sodium alginate, glycylglutamic acid, proline, and *cis*-resveratrol. KEGG pathway analysis revealed that up-regulated metabolites were enriched in the d-glutamine and d-glutamate metabolism pathways. In contrast, down-regulated metabolites were enriched in the primary bile acid (BA) biosynthesis, arginine biosynthesis, and glutathione metabolism pathways (Fig. 6B). These findings were consistent with our metabolic flux data, which indicated an increase in BA biosynthesis and BA recycling following acupuncture or sham-acupuncture intervention (Fig. 6C). Furthermore, BA biosynthesis was enriched in cMonocytes and nMonocytes (Fig. 6C).

### Gut microbiome analysis revealed acupuncture-induced enrichment of *Bilophila*

To characterize differences in the gut microbiome, we employed WMS with fecal samples from 23 patients in the acupuncture group and 21 patients in the sham-acupuncture group, both before and after the trial. We identified 20,399 microbes at the species level. Using conventional differential analysis, we identified 51 and 101 differential species in the comparisons of Acu_post vs. Acu_pre and Acu_post vs. Sham_post, respectively, with 0 overlapping metabolites between the 2 comparisons (Tables S10 and S11, *P* < 0.05).

**Fig. 7.**
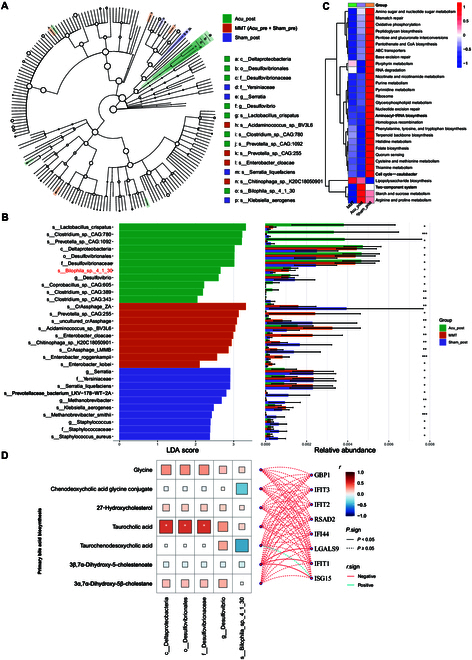
Fecal metagenomic profiles depicting the effect of acupuncture on methadone reduction. (A) Differential-species cladogram based on linear discriminant analysis effect size (LEfSe) analysis: green represents the Acu_post group, purple represents the Sham_post group, and orange represents the MMT group (Acu_pre + Sham_pre). (B) Linear discriminant analysis (LDA) score plot for differentially abundant species and an abundance plot based on LEfSe analysis. *P < 0.05, **P < 0.01, and ***P < 0.001, as determined using Kruskal–Wallis tests. (C) Heatmaps of KEGG pathway enrichment analysis. (D) Spearman correlations in the Acu_post group between the differentially produced metabolites in the primary bile acid biosynthesis pathway, *Bilophila*-related taxa, and interferon-stimulated genes (ISGs).

Discriminant analysis of differentially abundant species among all groups (Acu_post vs. Sham_post vs. MMT [Acu_pre and Sham_pre]) revealed that *Bilophila_sp._4_1_30* and its related taxa, including *Desulfovibrio*, Desulfovibrionaceae, Desulfovibrionales, and Deltaproteobacteria, exhibited a high discriminative weight for the Acu_post group and were highly abundant in this group (Fig. 7A and B and Table S9, *P* < 0.05). Additionally, *Lactobacillus crispatus* showed increased abundance following acupuncture. We also observed that acupuncture treatment led to enrichment in several KEGG pathway terms, such as lipopolysaccharide biosynthesis, the 2-component system, starch and sucrose metabolism, and arginine and proline metabolism (Fig. 7C).

To explore acupuncture-related associations across different omics platforms, we performed Spearman correlations with the Acu_post group to distinguish differentially abundant metabolites associated with the primary BA biosynthesis pathway and *Bilophila_sp._4_1_30*-related taxa, as well as ISGs (Fig. 7D). Statistically significant correlations (*P* < 0.05) were observed between taurocholic acid and Desulfovibrionaceae, Desulfovibrionales, and Deltaproteobacteria. Taurochenodesoxycholic acid and ISGs (except for *ISG15*) correlated negatively with differentially abundant metabolites in the primary BA biosynthesis pathway. Notably, the correlations between 3β,7α-dihydroxy-5-cholestenoate and both *ISG15* and *IFIT1* were statistically significant.

### BA treatment reduced galectin-9 expression in monocyte THP-1 cells

**Fig. 8.**
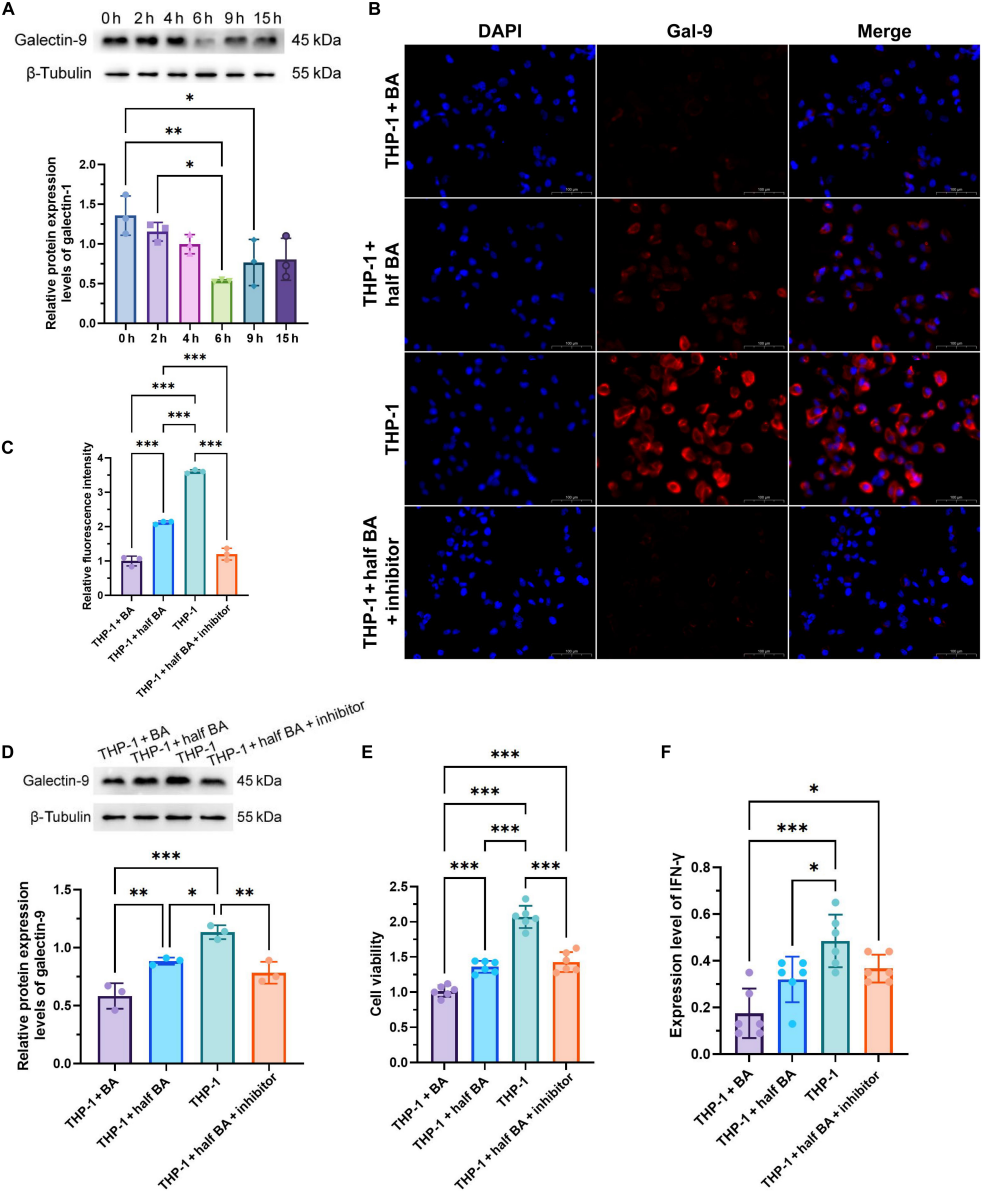
Effects of bile acid treatment in THP-1 monocytes. (A) Time-course analysis of galectin-9 protein levels: western blot analysis (top) and quantitative densitometry (bottom). (B) Immunofluorescence staining of galectin-9 (red) and 4′,6-diamidino-2-phenylindole (DAPI; blue). (C) Quantification of galectin-9 fluorescence intensity from panel (B). (D) Western blot (top) and densitometric (bottom) analysis of galectin-9 protein expression under the same treatment conditions as in panel (B): β-tubulin was used as a loading control. (E) Cell viability assessed by Cell Counting Kit-8 (CCK-8) assay. (F) IFN-γ expression levels measured by enzyme-linked immunosorbent assay (ELISA). *P < 0.05; **P < 0.01; ***P < 0.001.

Furthermore, we employed BA and half-BA-treated THP-1 monocyte models as in vitro systems to simulate the potential modulatory effects of acupuncture on BA metabolism. A time-course experiment revealed a time-dependent reduction in galectin-9 expression, with the most pronounced down-regulation observed at 6 h post-treatment (Fig. 8A). Accordingly, this 6-h timepoint was selected for all subsequent experiments.

Immunofluorescence staining demonstrated a marked down-regulation of galectin-9 expression in THP-1 cells following BA treatment. Notably, treatment with half-dose BA partially reversed this down-regulation, while the combination of half-dose BA and a galectin-9 inhibitor further suppressed galectin-9 expression to levels comparable with that for full-dose BA treatment (Fig. 8B and C). Western blot analysis further confirmed these trends (Fig. 8D).

Cell Counting Kit-8 (CCK-8) assays revealed that BA treatment significantly reduced cell viability compared with that of the control group (Fig. 8E), with a more pronounced decrease observed in the standard BA concentration group (80 μM) compared to the half-dose group (40 μM). Enzyme-linked immunosorbent assay (ELISA) results showed that IFN-γ levels were significantly decreased in the THP-1 + BA, THP-1 + half BA, and THP-1 + half BA + inhibitor groups relative to that in the control group (Fig. 8F). The THP-1 + half BA group exhibited a partial reversal trend in IFN-γ suppression compared to the THP-1 + BA group.

## Discussion

In our previously published randomized clinical trial, we demonstrated that acupuncture markedly reduced methadone dosage and opioid cravings compared to sham acupuncture, underscoring its potential as an adjunctive therapy for OUD [24]. Elucidating the underlying mechanisms is crucial to justifying its use and enhancing patient acceptance. Opioids cause immunosuppression, gut microbiota dysbiosis, metabolic disruptions, and persistent neurochemical disturbances [9]. Acupuncture can bidirectionally regulate the immune system and intestinal microbiota; however, its precise action mechanisms in treating opioid addiction remain unclear [18]. To date, scRNA-seq has not been utilized to investigate the clinical effects of acupuncture. Therefore, we employed scRNA-seq and multi-omics approaches to elucidate the potential mechanisms underlying the effect of acupuncture on methadone reduction in a randomized placebo-controlled clinical trial, followed by comprehensive omics research.

One of our most noteworthy findings was the correlation between cMonocytes and clinical acupuncture responses to methadone reduction. The biological significance of monocytes was evident from the results of the Augur model, enrichment analyses, cell communication analyses, clinical response analyses, and pseudotime trajectory analysis. Previous findings with human PBMCs from individuals with OUDs suggested that opioid exposure suppressed the antiviral gene program in naive monocytes [12]. Notably, various cell types were enriched for antiviral response pathways after acupuncture, including cMonocytes, CD8+NKT cells, and naive CD4+ T cells (Fig. 1C). Our pseudotime trajectory analysis indicated that the Acu_post group exhibited 2 specific temporal stages, potentially representing cell populations with acupuncture-specific effects (Fig. 4). Among these cell populations, we identified high expression levels of ISGs, such as *IFI44*, *IFIT2*, *IFIT1*, *ISG15*, *IFIT3*, *GBP1*, and *RSAD2*, at the end of the pseudotime trajectory. ISGs are a group of genes that are up-regulated in response to the presence of interferons, a type of cytokine [30]. Insufficient ISG responses can lead to increased susceptibility to viral infections, which may partly explain the higher susceptibility to viral infections observed in opioid users in epidemiological studies [31]. Acupuncture can up-regulate ISGs, suggesting that it was associated with antiviral defense responses.

Additionally, we confirmed that acupuncture enhanced intercellular communication (Fig. 2A and B). We compared communication probabilities and observed that ligand–receptor pairs (LGALS9–CD44 and LGALS9–CD45) mediated communications between cMonocytes and each cell cluster (Fig. 2C and Fig. S4). LGALS9, also known as galectin-9, is a member of the galectin protein family. The galectin family comprises carbohydrate-binding proteins that regulate immune responses, apoptosis, and their implications in diseases such as viral infections [32]. Galectin-9 binds to receptors on T-cell surfaces, primarily interacting with T-cell immunoglobulin and mucin domain-3 receptors, thereby influencing the balance of immune responses [33]. Galectin-9 also participates in antiviral immunity, for example, by regulating HIV infection processes [34]. Moreover, morphine inhibited inflammatory cytokine synthesis and galectin release, leading to reduced neutrophil recruitment in a mouse model of *Streptococcus pneumoniae* infection, increased pneumococcal bacterial loads in lung tissues, and systemic disease [35]. However, for technical reasons, we did not include neutrophils in our scRNA-seq analysis. However, in our total RNA sequencing (without cell sorting), the acupuncture effect-related gene set was enriched for the neutrophil extracellular trap formation pathway (Fig. 5C). This finding implies that up-regulating galectin-9 through acupuncture may promote neutrophil recruitment to infection sites, which may offer therapeutic potential.

The results of our metabolomics analysis indicated that the metabolites down-regulated by acupuncture were enriched for components of the BA biosynthesis pathway (Fig. 6B). This observation was corroborated by our metabolic flux analysis of the bulk RNA-seq data (Fig. 6C). The gut microbiota disruption and bile dysregulation caused by opioids lead to an impaired gut barrier and chronic systemic inflammation [36]. Chronic morphine treatment caused a marked shift in gut microbial composition, characterized by an increase in gram-positive pathogens and a decrease in bile-deconjugating bacterial genera, such as *Roseburia* and *Bilophila* [37]. Acupuncture and moxibustion positively regulate BA metabolism [38,39]. Lin et al. [40] found that herb-partitioned moxibustion treatment in rats with irritable bowel syndrome markedly improved the imbalance in BA metabolism, which mainly manifested in reduced BA transformation and reabsorption. Lee et al. [41] also reported that manual acupuncture can potentially decrease BA deposition, which may be associated with reduced activation of spinal microglia. A study of the gut microbiome–metabolite–brain axis suggests that BA modulation may be a promising intervention for treating neurological dysfunction [42]. Notably, we observed that the gut abundances of *Bilophila_sp._4_1_30* and its related taxa, including Desulfovibrionaceae, Desulfovibrionales, and Deltaproteobacteria, increased after acupuncture, based on microbiome analysis (Fig. 7A and B). In the Acu_post group, the abundance of taurocholic acid correlated with those of Desulfovibrionaceae, Desulfovibrionales, and Deltaproteobacteria (Fig. 7C). ISGs correlated negatively with differentially abundant metabolites in the primary BA biosynthesis pathway. *Bilophila* can metabolize the BA taurocholic acid and produce hydrogen sulfide (H_2_S) as a metabolite [43]. As a “Janus-faced metabolite”, H_2_S has the ability to function as an antioxidant within cells, a signaling molecule, or a source of mitochondrial energy, potentially providing positive effects on the host [44]. These findings suggest that acupuncture could potentially treat intestinal microbial disruption caused by OUDs by up-regulating *Bilophila* and BA metabolism.

Our in vitro findings further support the hypothesis that BA-mediated modulation of immune responses may underlie, at least in part, the biological effects observed with acupuncture in MMT patients. Specifically, we demonstrated that cholic acid treatment significantly down-regulated galectin-9 and IFN-γ expression in THP-1 monocytes, consistent with the immunosuppressive state observed in untreated MMT. Interestingly, treatment with half the standard concentration of BA (simulating acupuncture-induced down-regulation of BA metabolism) partially reversed the suppression of galectin-9 and IFN-γ. These results suggest that acupuncture may reverse opioid-induced immunosuppression by remodeling the BA–galectin–interferon axis. In the THP-1 + half BA + galectin-9 inhibitor group, galectin-9 protein expression showed a downward trend, but the difference was not statistically significant, and no significant changes were observed in IFN-γ expression or cell viability. The limited response may reflect that standard THP-1 culture conditions cannot fully mimic the immunosuppressed environment of MMT patients. Moreover, there is currently a lack of a selective galectin-9 inhibitor. The compound used in this experiment is a broad-spectrum galectin inhibitor with a stronger activity against galectin-3 and a lower specificity for galectin-9 [45,46]. Future studies using patient-derived immune cells and more selective inhibitors will be essential to clarify the specific role of galectin-9 in this regulatory axis.

The strength of this study lies in the combined use of multi-omics technologies, with results that corroborated and complemented each other. This study has some limitations: Due to the unique characteristics of the study population and challenges in clinical sample collection, the sample size for omics analysis was limited, which may reduce the generalizability of the findings. The omics subset was selected solely based on sample completeness—not on treatment response—which minimizes the risk of selection bias. Most of our findings are based on clinical samples; we cannot conclusively establish a causal link between acupuncture and the observed biological changes in patients. Although Augur and Scissor are powerful tools for linking cell-level features with clinical phenotypes and have shown strong performance in case–control studies [47,48], their use in randomized controlled trials is still emerging. Their performance may be affected by batch effects, sample size, and phenotype definition, so results should be interpreted with caution and ideally supported by complementary analyses or independent validation. The clinical sample was predominantly male, and the small number of female participants limited our ability to draw sex-specific conclusions. Future studies with more balanced sex representation are needed to examine potential sex-related differences in response to acupuncture. Despite rigorous control of baseline characteristics, unmeasured factors such as dietary patterns, smoking status, and micronutrient intake may have introduced residual confounding. Given the influence of environmental factors and lifestyle on gut microbiota composition, larger population-based studies with rigorous control of confounding variables are crucial to comprehensively evaluate the broader efficacy of acupuncture interventions in MMT. Constructing the omics libraries consumed a substantial amount of clinical samples, which limited the availability of material for downstream experimental validation. Although the in vitro experiments using THP-1 monocytes provided mechanistic insights, they cannot fully recapitulate the complexity of in vivo immune regulation. Future studies using primary patient-derived immune cells and in vivo animal models are necessary.

**Fig. 9.**
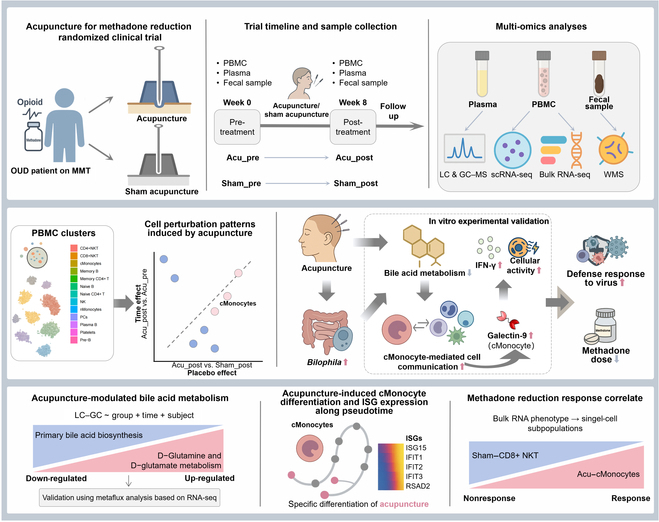
Schematic overview of the current study.

Collectively, our integrative analysis strategy connected clinical outcomes with immune, metabolic, and microbial features, providing a clinically relevant perspective on the biological effects of acupuncture. (Fig. 9). These findings highlight the efficacy of acupuncture in reducing methadone dose and its association with cMonocytes. Furthermore, the study identifies various acupuncture effect characteristics and their correlation. Acupuncture may ameliorate intestinal microbial disruptions caused by OUD by up-regulating *Bilophila* and modulating BA metabolism, suggesting a potential optimization of acupuncture benefits in the context of methadone reduction. Although these findings are promising, further in-depth and larger-scale studies are warranted to validate our results and explore the specific cell subclusters associated with treatment responses to acupuncture.

## Methods

### Trial design

As described in a previous study [24], the trial protocol was approved by the Ethics Committee of the Panyu Hospital of Traditional Chinese Medicine and was overseen by an independent trial steering committee (2022029). Study participants were informed of all details of the study, and each participant provided written informed consent. The trial protocol is available in Supplementary Materials.

The multicenter, patient-blinded, parallel-arm, 1:1 randomized clinical trial compared acupuncture with sham acupuncture for enhancing methadone reduction in patients undergoing MMT, with a 21-week trial period including baseline assessment, intervention, and follow-up phases. Participants included individuals aged 18 to 65 years receiving MMT and meeting DSM-5 criteria for OUDs [49], requiring methadone tapering. Among the participants enrolled, multi-omics profiling was conducted in a subset who completed the intervention; provided qualified paired biological samples for at least one of PBMCs, stool, or plasma; and gave informed consent for intensive biological sampling. The primary clinical outcome measured was the rate of methadone dose reduction, quantified as the proportion of participants whose daily dose of methadone decreased by ≥20% relative to baseline after 8 weeks of intervention. Patients who achieved a methadone reduction of ≥20% were identified as responders; otherwise, they are considered nonresponders. Detailed descriptions of trial design, participants, interventions, and outcomes are provided in the Supplementary Methods.

### Sample collection and processing specifications

Blood and fecal samples were collected at baseline and at the end of treatment (Fig. 1A). PBMCs were isolated for scRNA-seq and bulk RNA-seq, plasma was collected for metabolomics analysis, and fecal samples were collected for metagenomics analysis. The procedures followed for sample preparation, quality control analysis, and data preprocessing are detailed in the Supplementary Methods.

### Bioinformatics analysis

PBMCs were processed for scRNA-seq using the DNBelab C Series Single-Cell Library Prep Set (MGI, China). Data processing and cell clustering were performed using Seurat [50]. The data from all groups were integrated using the SCTransform function. The cell types were annotated using known markers and the reference database [51]. GO and KEGG enrichment analyses were performed by clusterProfiler [52]. The Augur model was used to construct a classification model for all cell types by random sampling between 2 groups (Acu_post vs. Acu_pre and Acu_post vs. Sham_post) [53]. Cell–cell interaction networks for all cell types in the acupuncture and sham-acupuncture groups (before and after the intervention) were predicted using CellChat [54]. Cell differentiation trajectories were inferred using Monocle2 [55].

For bulk RNA-seq, read counts were normalized, followed by differential expression analysis using DESeq2 [56]. Genes exhibiting |log_2_FC| > 0.5 and adjusted *P* values <0.05 were classified as DEGs. WGCNA was performed to identify gene modules specifically associated with the acupuncture intervention [57].

For LC–MS/MS- and GC–MS-based metabolic profiling, linear models were used to compare longitudinal differences between the acupuncture and sham-acupuncture groups using the time of treatment and the subject group as covariates. Longitudinally differential metabolites were selected with adjusted *P* values of < 0.05. Enrichment analysis was performed using MetaboAnalyst [58].

For the WMS data, we obtained the taxonomies of species from the corresponding taxonomy database of the National Center for Biotechnology Information Non-Redundant Protein Database Library, and abundance statistics were calculated to species levels. Linear discriminant analysis effect size was used to identify key biomarkers.

For integrative analysis, Scissor was used to identify bulk phenotype-associated cell subpopulations [59]. To quantify metabolic activities, we performed flux-balance analysis to derive metabolic pathways for scRNA-seq and bulk RNA-seq datasets using METAFlux [60]. Detailed descriptions are presented in the Supplementary Methods.

### In vitro experimental validation

#### Cell culture

THP-1 cells were purchased from Wuhan PriCells Life Technology Co., Ltd. Cells were cultured in RPMI-1640 medium (Gibco, USA) supplemented with 10% fetal bovine serum (Gibco, USA ), 0.05 mM β-mercaptoethanol, 100 U/ml penicillin, and 50 U/ml streptomycin at 37 °C in a humidified atmosphere of 5% CO_2_. When cell confluence reached over 80%, cells were digested with 0.25% trypsin for 1 min, followed by neutralization with complete medium. Cells were then seeded into 6-well plates for further experiments.

#### Experimental design

To investigate the regulatory effects of cholic acid (81-25-4, MCE, USA) on THP-1 cells, we first conducted a time-course experiment to determine the optimal treatment duration. THP-1 cells were exposed to 80 μM cholic acid for 0, 2, 4, 6, 9, and 15 h, respectively [61–63].

Following the determination of optimal treatment time, cells were divided into 4 experimental groups for comparative analysis: (a) THP-1 + BA group: treated with 80 μM cholic acid for 6 h, representing the pathological BA state associated with MMT; (b) THP-1 + half BA group: treated with 40 μM cholic acid for 6 h to evaluate the effect of a reduced concentration, representing acupuncture-induced down-regulation of BA metabolism; (c) THP-1 group: treated with equal volumes of culture medium without cholic acid for 6 h as the baseline control; and (d) THP-1 + half BA + inhibitor group: treated with 40 μM cholic acid and 37 nM galectin inhibitor (1978336-61-6, MCE, USA) for 6 h to assess the potential involvement of galectin signaling.

#### Western blot

Cells were lysed using radioimmunoprecipitation assay buffer supplemented with phenylmethylsulfonyl fluoride and protease inhibitors. Protein concentration was determined using the bicinchoninic acid assay. Equal amounts of protein were separated by sodium dodecyl sulfate–polyacrylamide gel electrophoresis and transferred to PVDF membranes (0.22 μm, Biodai, China). Membranes were blocked with blocking buffer and incubated with primary antibodies against galectin-9 (Proteintech, China) and β-tubulin (CST, USA) at 4 °C overnight. After washing, membranes were incubated with horseradish peroxidase-conjugated secondary antibodies at room temperature for 2 h. Protein bands were visualized using an enhanced chemiluminescence system (Tanon 5200) and quantified using the ImageJ software.

#### CCK-8 cell viability assay

THP-1 cells were seeded in 96-well plates at a density of 2,000 cells per well and incubated at 37 °C for 6 h. Cells were treated according to the experimental groups. After 6 h of treatment, 10 μl of CCK-8 reagent (Proteintech, China) was added to each well, and cells were incubated for an additional 2 h. The absorbance at 450 nm was measured using a microplate reader.

#### Immunofluorescence staining

THP-1 cells were seeded onto 6-well plates, fixed with 4% paraformaldehyde for 20 min, and permeabilized with 0.25% Triton X-100 for 20 min. After blocking with goat serum, cells were incubated overnight with primary antibodies at 4 °C. The next day, cells were washed and incubated with fluorescent secondary antibodies at room temperature for 1 h, followed by nuclear staining with 4′,6-diamidino-2-phenylindole (Beyotime, China). Fluorescence images were captured using a fluorescence microscope (BZ-X800, Keyence, Japan), and fluorescence intensity was analyzed using the ImageJ software.

#### Enzyme-linked immunosorbent assay

Supernatants from treated cells were collected, centrifuged at 1,000 × g for 20 min, and analyzed using Human IFN-γ High Sensitivity ELISA Kit (Liankebio, China). The absorbance at 450 nm was measured using a microplate reader.

### Statistical analysis

All statistical analyses were performed using the R software (v4.3.1) and GraphPad Prism (v9.5.1). Categorical variables were described as numbers and percentages. Group comparisons were performed using the chi-square test or Fisher’s exact test. Continuous variables were reported as mean with standard deviation or median and interquartile range. Two-group comparisons were analyzed using the Student *t* test or Mann–Whitney *U* test, while multiple-group comparisons were performed using one-way analysis of variance followed by Tukey’s post hoc test for pairwise comparisons. Spearman’s rank correlation coefficient was used for correlation analysis to assess the nonlinear relationships between variables. Correlation analyses were conducted using only samples with complete data; no imputation was applied. All statistical tests were 2-sided at the 5% significance level.

## Data Availability

The omics raw datasets, processed summary results, and analysis scripts used for statistical analysis and multi-omics integration are available to qualified researchers upon reasonable request from corresponding author Prof. Liming Lu (lulimingleon@126.com). These data will be available for a period of 6 months to 3 years after publication. Data requests require a methodologically sound proposal as well as a data access agreement and approval by the local ethics committee.

## Supplementary Materials

Supplementary 1 Figs. S1 to S6 Tables S1 to S11 Supplementary Methods Trial Protocol
