# Supplementary material for: Single-Cell RNA Transcriptomics and Multi-omics Analyses Reveal the Clinical Effects of Acupuncture on Methadone Reduction
Source: Research (Wash D C). 2025 Jun 24;8:0741. doi: 10.34133/research.0741 (PMC12187353; doi:10.34133/research.0741)
Supplement: Supplementary 1 — Figs. S1 to S6 Tables S1 to S11 Supplementary Methods Trial Protocol [file research.0741.f1.zip › Supplementary Figures S1-S6.docx]

**
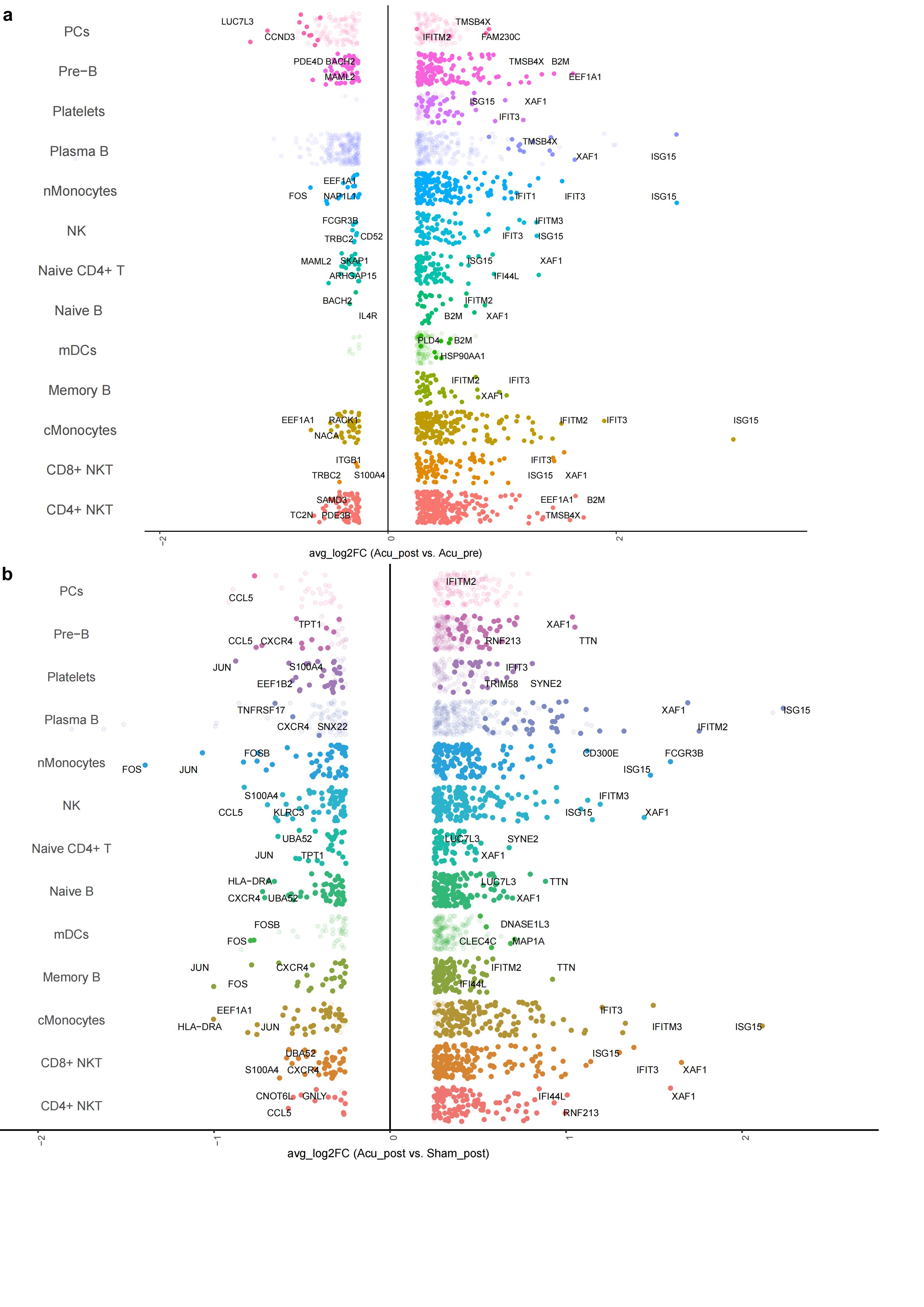
Fig. S1** (related to Fig. 1) Volcano plot showing gene-expression fold changes (FCs) (log_2_ scale) for each cell cluster. **a** Within-group variation (Acu_post vs. Acu_pre). **b** Between-group variation (Acu_post vs. Sham_post).

**
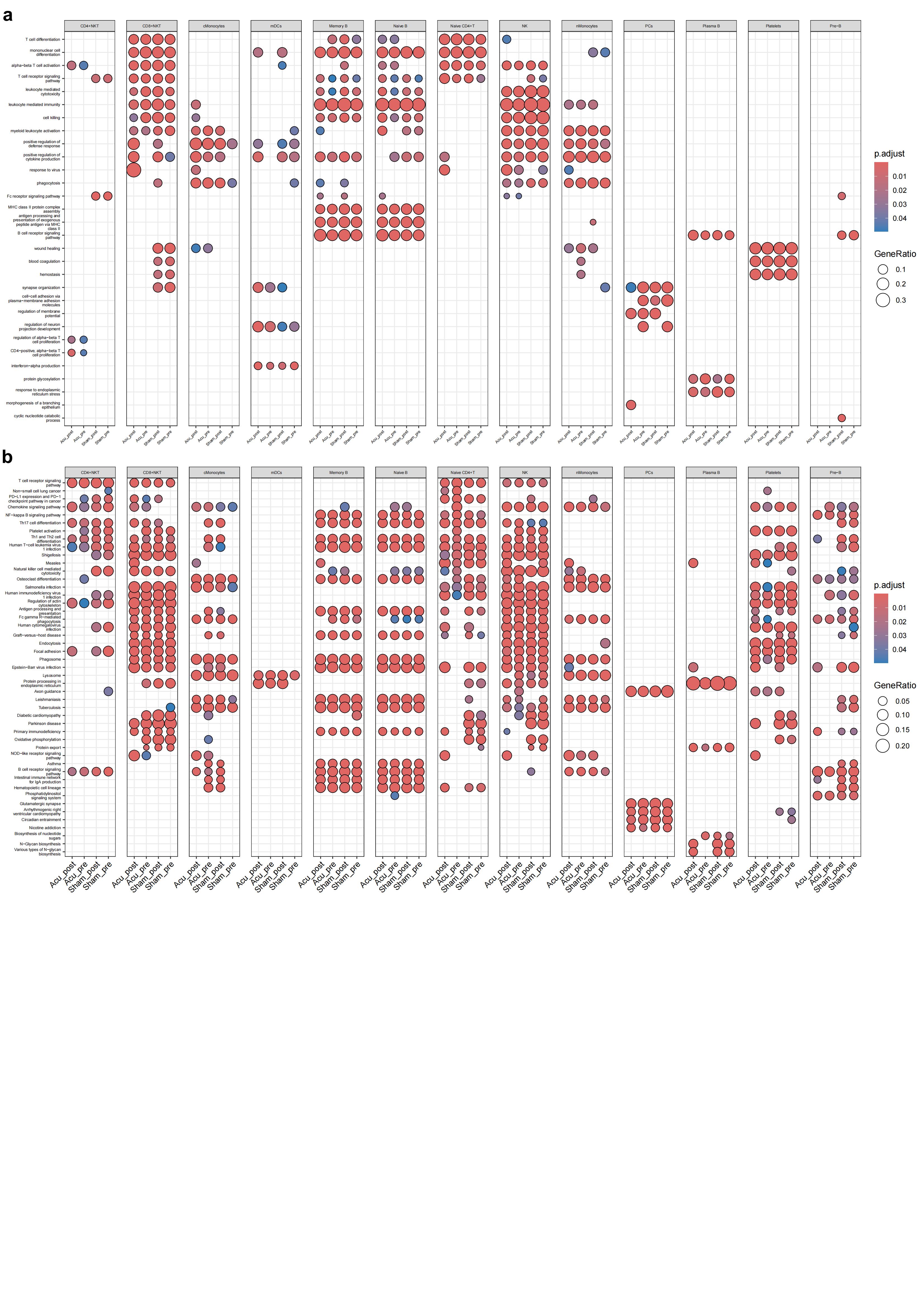
Fig. S2** (related to Fig. 1) Enrichment analysis of marker genes from each group and cell cluster. **a** GO-term enrichment analysis of the top 100 marker genes. **b** KEGG pathway-enrichment analysis of the top 1,000 marker genes.

**
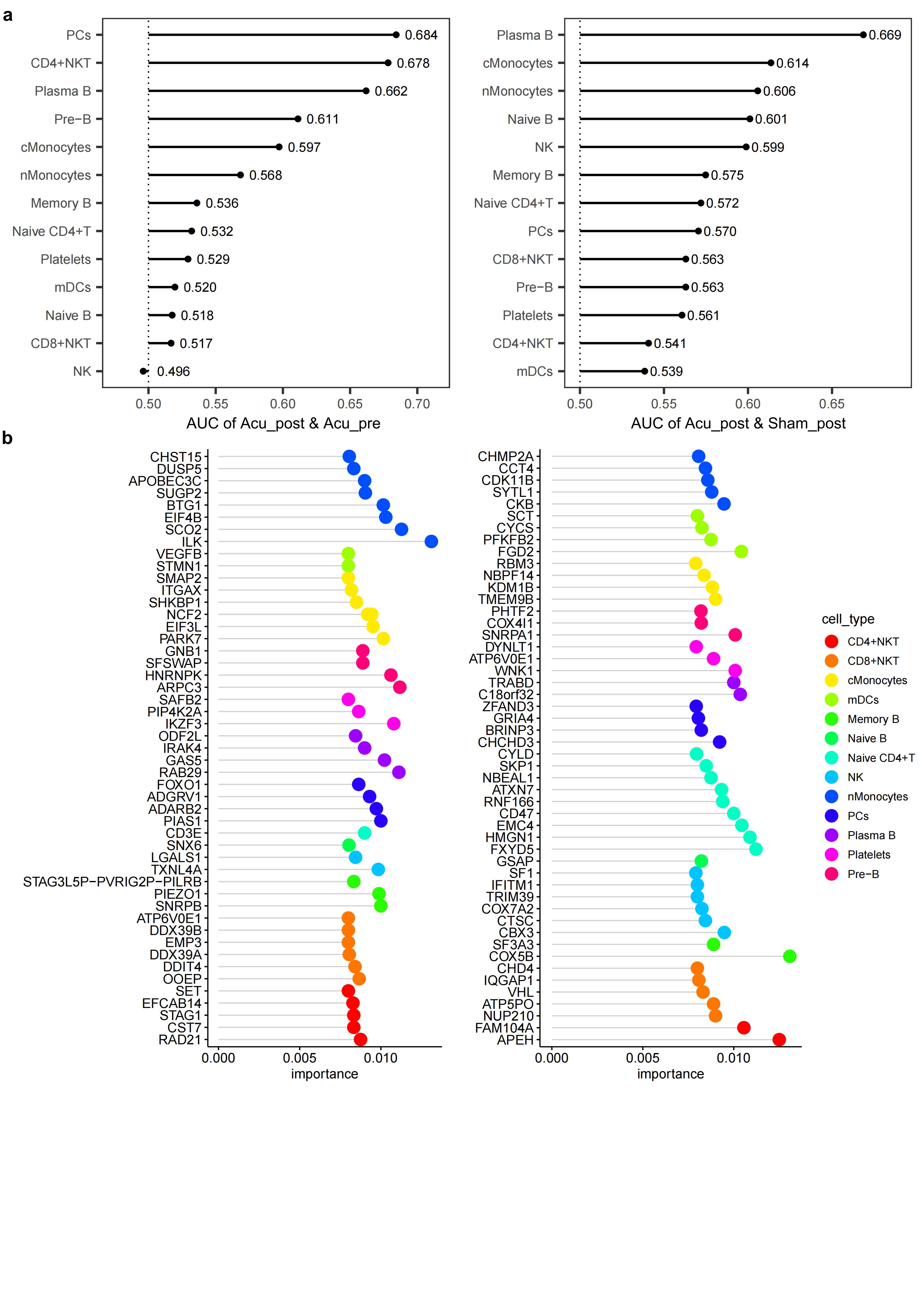
Fig. S3** (related to Fig. 1) Augur model. **a** AUC reflect the degrees of differences in cell types between groups. The left panels represent Acu_post vs. Acu_pre comparisons, and the right panels represent Acu_post vs. Sham_post comparisons. **b** Gene importance scores for each cell cluster. The left panels represent the Acu_post vs. Acu_pre comparison, and the right panels represent the Acu_post vs. Sham_post comparison.

**
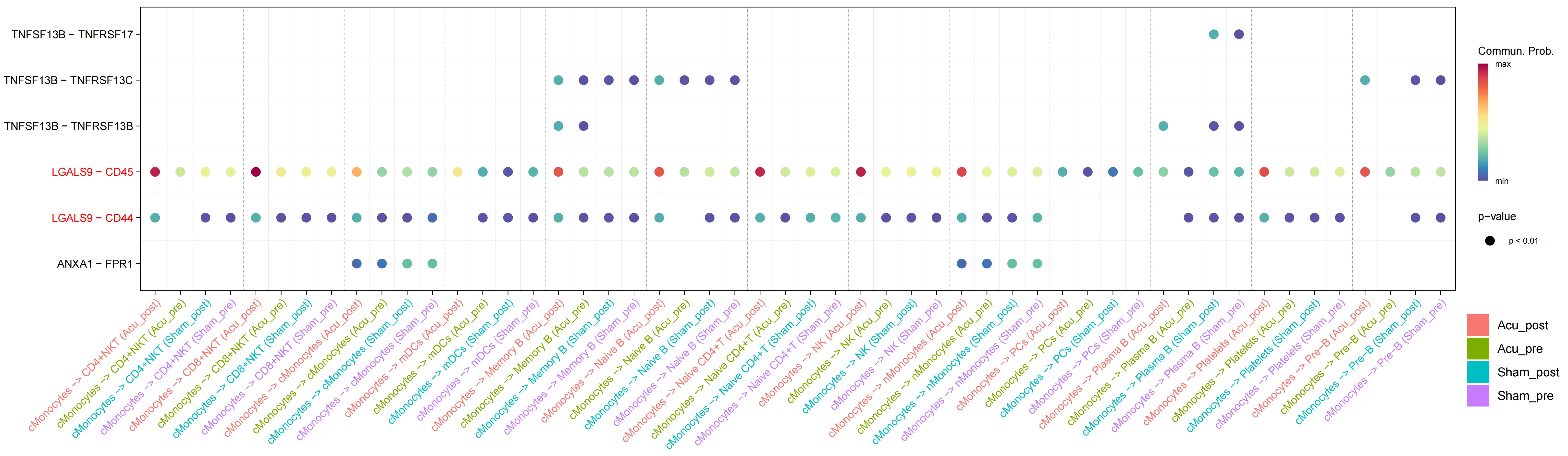
Fig. S4** (related to Fig. 2) Dot plot showing the ligand–receptor-mediated communication probabilities between cMonocytes and other cell clusters.

**
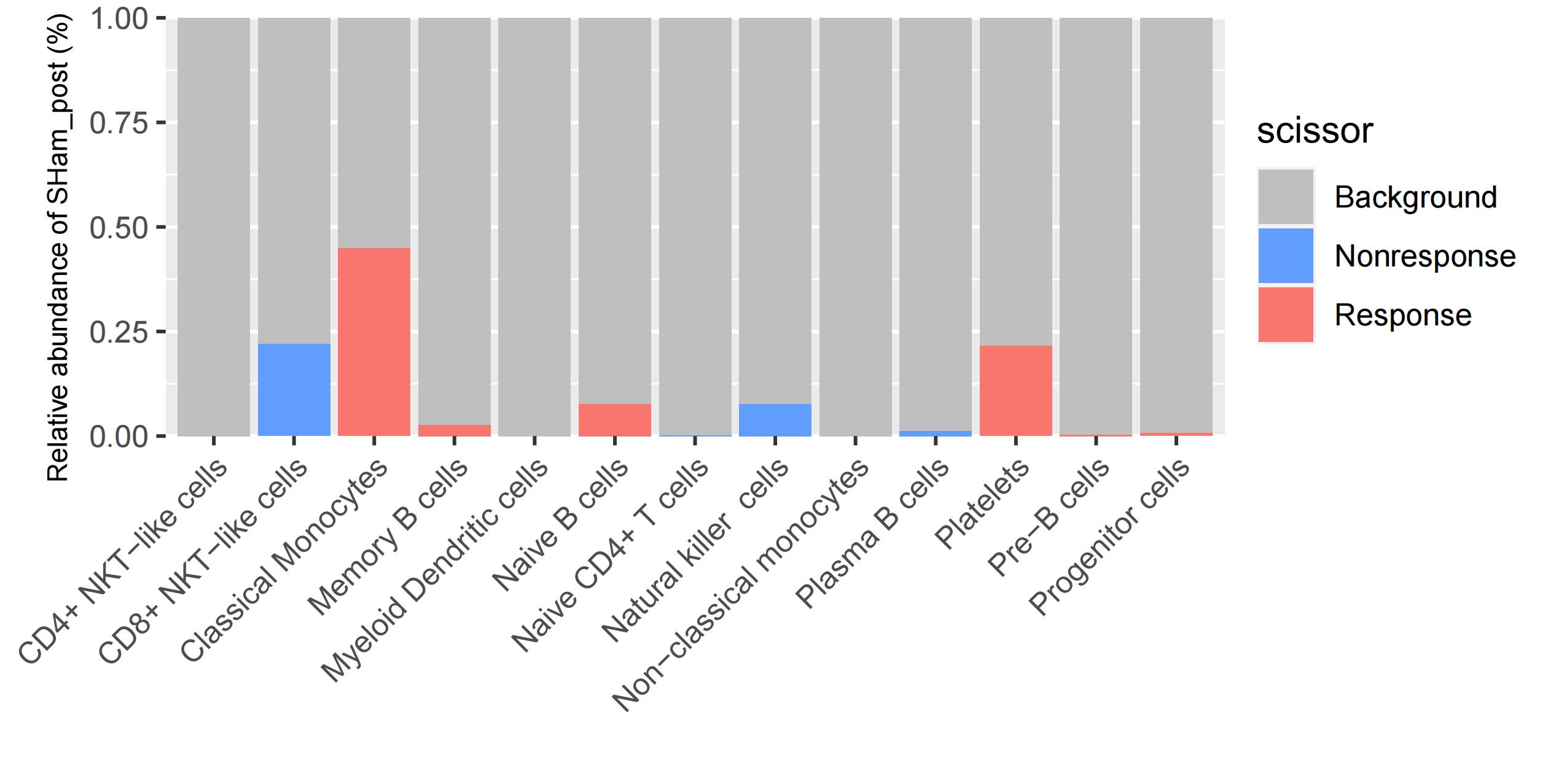
Fig. S5** (related to Fig. 3) Bar plot showing the proportional fractions of the different identified cell types in the Sham_post group.

**
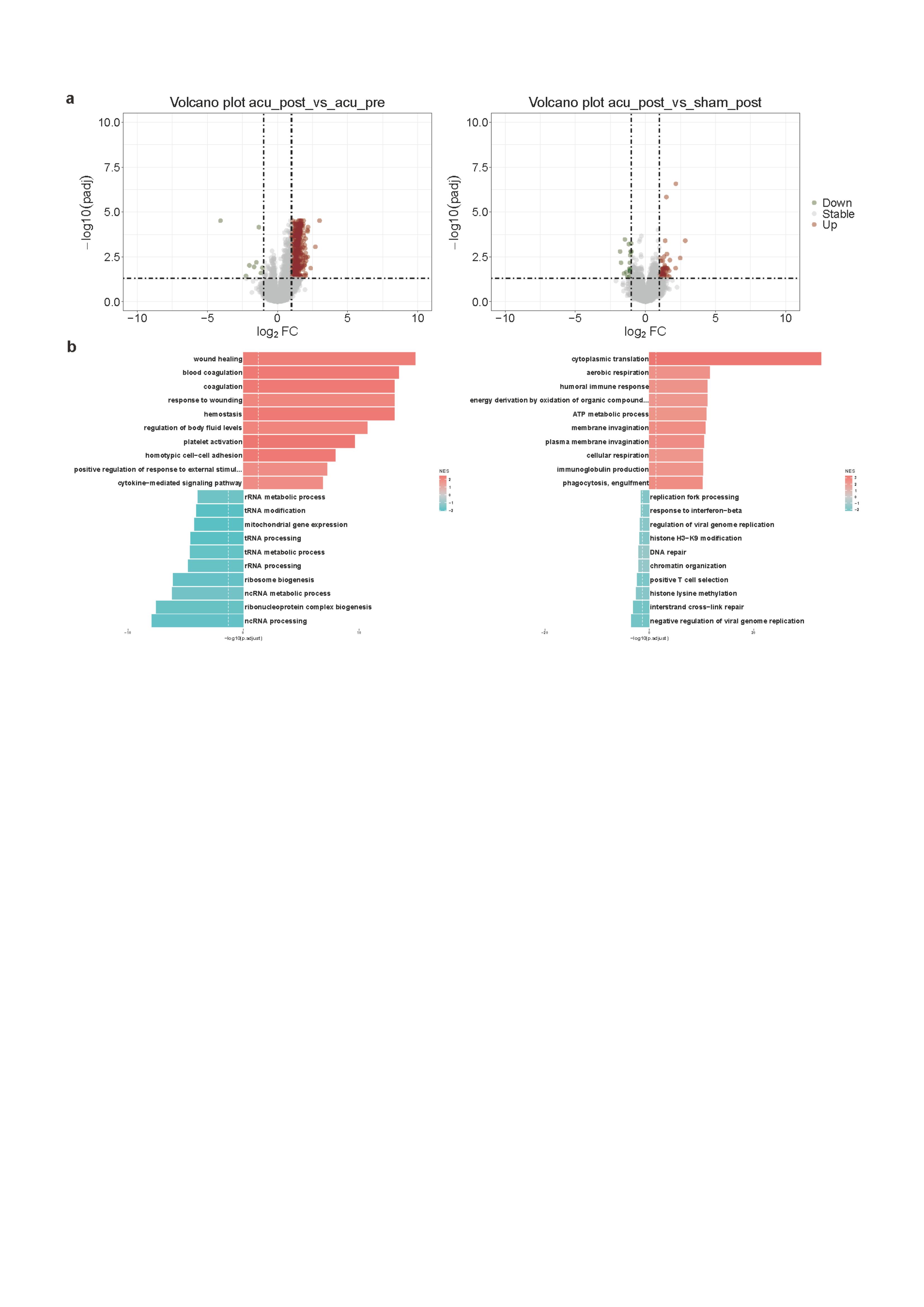
Fig. S6** (related to Fig. 5) Differential expression analysis for inter-group or intra-group comparisons with the bulk RNA-seq data. **a** Volcano plot showing gene-expression FCs (log_2_ scale) for the Acu_post vs. Acu_pre (left panel) and Acu_post vs. Sham_post (right panel) comparisons. **b** GO-term enrichment analysis of DEGs for the Acu_post vs. Acu_pre (left panel) and Acu_post vs. Sham_post (right panel) comparisons. Rightward movement represents upregulation, and leftward movement represents downregulation.
