# Supplementary material for: Single-Cell RNA Transcriptomics and Multi-omics Analyses Reveal the Clinical Effects of Acupuncture on Methadone Reduction
Source: Research (Wash D C). 2025 Jun 24;8:0741. doi: 10.34133/research.0741 (PMC12187353; doi:10.34133/research.0741)
Supplement: Supplementary 1 — Figs. S1 to S6 Tables S1 to S11 Supplementary Methods Trial Protocol [file research.0741.f1.zip › Supplementary Methods.pdf]

Supplementary Methods

Trial Design

As described in the previous study, the trial protocol was approved by the Ethics Committee of the Panyu Hospital of Traditional Chinese Medicine and was overseen by an independent trial steering committee (2022029). Study participants were informed of all details of the study and each participant provided written informed consent. The trial protocol is available in Supplementary Materials.

This clinical trial was designed as a multicenter, patient-blinded, parallel-arm, 1:1 randomized clinical trial (RCT) to test the superiority of acupuncture, compared with sham acupuncture, for improving methadone reduction outcomes in MMT clients (Fig. 1a). Enrolment began April 1, 2022, and ended November 30, 2022 at 6 MMT clinics in China (https://www.chictr.org.cn: ChiCTR2200058123). The total trial period was 21 weeks, including one week of baseline assessment, 8 weeks of intervention after randomization, and 12 weeks of follow-up post-treatment. Final follow-up occurred April 30, 2023.

Participants

Participants were MMT clients fulfilled the diagnostic criteria for OUDs as defined by the fifth edition of the Diagnostic and Statistical Manual of Mental Disorders (DSM-5) [1].

The inclusion criteria were males or females aged 18-65 years, having received MMT for more than 6 weeks, having demand for methadone tapering, and not having

received any acupuncture therapy during the previous 3 months. Subjects were excluded if they: a. had serious heart, liver, lung, or kidney diseases; b. had syphilis or AIDS; c. had a severe digestive disease or malnutrition; d. severe primary hematological disorders; e. had a history of mental illness other than drug dependence; f. had received other treatment that may affect the efficacy evaluation of the present intervention; g. had an infection, inflammation, scar, or injury close to the site of the selected acupoints; h. were pregnant or were planning pregnancy.

Among the participants enrolled, multi-omics profiling was conducted in a subset who: a. completed the intervention; b. provided qualified paired biological samples for at least one of PBMCs, stool, or plasma; c. gave informed consent for intensive biological sampling.

Interventions

Subjects assigned to each group received acupuncture or sham acupuncture for 30 min per session. A total 24 sessions, 3 sessions per week were carried out, followed by a 12-week follow-up. Manual acupuncture will be applied at acupoints that belong to Jin's three-needle acupuncture (JTN), including Sishen-I (GV21), Sishen-II (GV19), Sishen-III, Sishen-IV, Dingshen-I, Dingshen-II, Dingshen-III, Shouzhi-I (HT7), Shouzhi-II (PC6) and Shouzhi-III (PC8). Details of acupuncture (location of acupuncture points, depth of insertion) are shown in Supplement 1. Over a 30-minute period, manual manipulation for each acupoint lasted 10 seconds and was repeated three times with intervals of 10 minutes. For sham acupuncture, non-penetrating sham acupuncture will be applied at the same acupoints as the acupuncture group. The

device has already been granted a patent by China's national intellectual property administration (No. ZL 202223328917.3). Participants were blinded to group and intervention by entering the needle through this acupuncture auxiliary device.

Outcomes

One primary outcome was the rate of methadone dose reduction, defined as the proportion of participants who achieved a reduction in methadone daily dose of ≥20% compared to baseline after 8 weeks intervention. Patients who achieved a methadone reduction of ≥20% were identified as responders, otherwise, they are considered non-responders.

Sample collections and processing specifications

Blood and fecal samples were collected at inclusion and end of treatment (8-week later) (Fig. 1a). PBMCs and plasma samples were isolated from patients using with EDTA-anticoagulant tubes. PBMCs were separated using lymphocyte separation solution (Solarbio Science & Technology Co. Beijing, China), and then stored with Serum-free cell cryopreservation solution (Solarbio Science & Technology Co. Beijing, China). Fecal samples were collected in sterile polypropylene conical tubes within 15 min of defecation. These specimens were stored at -80℃ for analysis. PBMCs were tests for scRNA-seq and bulk RNA-seq; plasma samples were tests for metabolomics profiles; fecal samples were tests for metagenomic profiles.

scRNA-seq analysis

PBMCs collected from patients were used for single-cell RNA sequencing. Live PBMCs were collected by magnetic bead purification (Miltenyi Biotech) before scRNA-seq was performed. Cells were resuspended with cell resuspension buffer at a viable cell concentration of 1,000 cells/μL. The DNBelab C Series Single-Cell Library Prep Set (MGI, China) was utilized for single-cell RNA-seq library preparation. In brief, single-cell suspensions were used for droplet generation, emulsion breakage, beads collection, reverse transcription, and cDNA amplification to generate barcoded libraries. The sequencing libraries were sequenced by the MGISEQ-T7 sequencer (MGI, China) for paired reads of 100 bp.

Single-cell RNA-seq data processing of raw FASTQ files (Alignment, Barcode Assignment, and UMI Counting) were transformed using DNBelab C Series scRNA analysis software (v1.0.1). Data from cells expressing <500 genes, containing >20% of mitochondrial gene reads and under 1300 unique molecular identifier (UMI) were categorized as low-quality and subsequently removed. Cell clustering was accomplished by the Seurat (v4.4.0) R package [2]. Data integration from all groups was conducted using the SCTransform function.

In order to identify different cell types clusters, differentially expressed genes were analyzed for each cluster and representative markers were annotated based on their expression. When annotating the cell type, we identified marker genes that are known to be specific to certain cell types according to immunology basics. We then used these marker genes to perform clustering analysis to identify different cell populations in the scRNA-seq data. We also used reference cell type databases such as

Sctype (https://sctype.app/) to annotate the cell types based on the marker genes expressed in our dataset [3].

GO and KEGG enrichment analyses were performed by clusterProfiler(v4.10.0) R package [4].

Use the Augur model to construct a classification model for all cell types by randomly sampling between two groups (Acu_post vs. Acu_pre and Acu_post vs. Sham_post), and then compare the results with experimental labels through the constructed model, and reflect the degree of difference of cell types between groups through the Area Under the Curve (AUC) values [5].

The cell-cell interaction network of all celltype in the acupuncture group and sham acupuncture group before and after the intervention were predicted using CellChat (v1.6.1) R package with default parameters [6]. Run CellChat on each dataset separately and then merge different CellChat objects together, then compared the total number of interactions and interaction strength of the inferred cell-cell communication networks. Finally, we identified the up-regulated and down-regulated signaling ligand-receptor pairs by comparing the communication probability.

Cell differentiation trajectories were inferred using the Monocle2 (v2.30.0) package [7]. Monocle introduces a method for temporally ordering cells by employing the Reversed Graph Embedding technique from the field of machine learning, a strategy known as pseudotime analysis. First, the newCellDataSet function is used to import the expression matrix, phenotype information, and clustering results created by Seurat, ensuring the continuity of the research. After performing data correction and

filtering, the setOrderingFilter function is used to arrange all cells in pseudotime based on the set of highly variable genes calculated by Seurat, obtaining the temporal information and cell stages of all cells. The differentialGeneTest function is used to calculate the highly variable genes that change with pseudotime.

Bulk RNA-seq analysis

Total RNA extraction from PBMCs was used TRIzol (Invitrogen) and Direct-zol RNA Miniprep (Zymo Research, Irvine, USA). A total amount of 0.2 μg of total RNA per sample was processed for preparing mRNA sequencing library using the VAHTS® Universal V8 RNA-seq Library Prep Kit for MGI (Vazyme, Nanjing, China) according to manufacturer's instruction. The library preparations were sequenced on MGISEQ-T7 and 150bp paired end reads were generated.

Low-quality bases and adapter sequences bases were trimmed using fastp (v0.23.0). The trimmed reads were aligned to the human genome assembly GRCh38 using Hisat2 (v2.2.1). Read counts were normalized and differential expression analysis between conditions were performed using DESeq2 (v1.40.2) R package [8]. Genes with $|log2FC| > 0.5$ and adjusted P-values < 0.05 were identified as DEGs.

The WGCNA (v1.72) R package was utilized to construct a weighted gene co-expression network and identify the gene modules specifically associated with acupuncture intervention [9]. This involved calculating pairwise correlations between genes, followed by the transformation of the correlation matrix into an adjacency matrix using a chosen soft thresholding power. The co-expression network was

clustered into distinct modules using hierarchical clustering algorithms. Modules are sets of genes that are highly interconnected, indicating potential functional relationships. The relationship between modules and groups was investigated. This involved correlating the module eigengenes with the trait of interest and performing statistical tests to determine the significance of the associations. Subsequently, the relationship between key module and group was determined using Pearson correlation analysis.

Plasma metabolomics profiles using LC-MS and GC-MS

For LC-MS-based metabolic profiling, 150 μL of thawed plasma were diluted with 600 μL of ce-cold mixture of methanol and acetonitrile (2/1, vol/vol, containing L-2-chlorophenylalanine at a concentration of 2 μg/mL), and the mixtures were vortexed for 1 min, and the whole samples were extracted by ultrasonic for 10 min in ice-water bath， stored at -40 °C for 120 min. The extract was centrifuged at 4°C (12,000 rpm) for 10 min, 150 μL of supernatant was filtered through 0.22 μm microfilters and transferred to LC vials.

For GC-MS-based metabolic profiling, 150 μL of thawed plasma were diluted with 600 μL of ce-cold mixture of methanol and acetonitrile (2/1, vol/vol, containing L-2-chlorophenylalanine at a concentration of 2 μg/mL), and the mixtures were vortexed for 1 min, and the whole samples were extracted by ultrasonic for 10 min in ice-water bath， stored at -40 °C for 30 min. The extract was centrifuged at 4°C (12,000 rpm) for 10 min, 150 μL of supernatant in a glass vial was dried in a freeze

concentration centrifugal dryer. And 80 μL of 15 mg/mL methoxylamine hydrochloride in pyridine was subsequently added. The resultant mixture was vortexed vigorously for 2 min and incubated at 37 °C for 60 min. 50 μL of BSTFA (with 1% TMCS) and 20 μL n-hexane were added into the mixture, which was vortexed vigorously for 2 min and then derivatized at 70 °C for 60 min. The samples were placed at ambient temperature for 30 min before GC-MS analysis.

Nontargeted LC-MS analysis was performed on a ACQUITY UPLC I-Class system (Waters Corporation, Milford, USA) equipped with an ACQUITY UPLC HSS T3 column (100 mm×2.1 mm, 1.8 um, Waters Corp., U.S.A.). The mobile phases A and B were water and acetonitrile, both containing 0.1% formic acid. The injection volume was set at 2 μL, and the flow rate was constant at 0.35 mL/min. Data acquisition was performed in full scan mode (m/z ranges from 100 to 1200) combined with MSE mode.

GC-MS metabolic profiling was performed on the Agilent 7890B/5977A Series Gas Chromatograph/Mass Selective Detector system, equipped with a DB-5MS capillary column (30 m × 0 25 mm × 0 .25 μm, Agilent J & W Scientific, Folsom, CA, USA). Helium (> 99.999%) was used as the carrier gas at a constant flow rate of 1 mL / min through the column. The injector temperature was maintained at 260 ℃. Injection volume was 1 μL by splitless mode. Mass spectrometric data was acquired in a full-scan mode (m/z 50-500).

The quality control (QC) sample was a mixture of equal amount of each sample involved, set to monitor the stability of both the LC-MS and the GC-MS system and

for signal correction. The blank samples were used to assess the cleanliness status of the instrument.

Raw data of LC-MS were processed by software Progenesis QI (V2.3, Nonlinear, Dynamics, Newcastle, UK). Raw data of GC-MS were imported into software MS-DIAL. After the data was normalized, redundancy removal and peak merging were conducted to obtain the data matrix. The Human Metabolome Database (HMDB), Lipidmaps (v2.3), Metlin, EMDB and PMDB were used to qualitative analysis. Variable Importance of Projection (VIP) values obtained from the Orthogonal Partial Least-Squares-Discriminant Analysis (OPLS-DA) model were used to rank the overall contribution of each variable to group discrimination. A two-tailed Student's T-test was further used to verify whether the metabolites of difference between groups were significant. Differential metabolites were selected with VIP values greater than 1.0 and *P*-values less than 0.05. Using linear models to compare the longitudinal differences between acupuncture and sham acupuncture, with covariates as Time and Object. Longitudinal differential metabolites were selected with adjusted *P*-values less than 0.05. Enrichment analysis was performed by MetaboAnalyst 5.0 platform (https://www.metaboanalyst.ca/) [10].

Fecal metagenomic profiles

Total DNA was isolated from fecal samples using a QIAamp® Fast DNA Stool Mini Kit (Qiagen, Hilden, Germany). Then the libraries were constructed using TruSeq Nano DNA LT Sample Prepararion Kit (Illumina, San Diego, CA, USA) according to

the manufacturer's instructions. The libraries were sequenced on llumina Novaseq 6000 platform and 150 bp paired-end reads were generated.

The taxonomy of the species was obtained as a result of the corresponding taxonomy database of the NCBI Non-Redundant Protein Database (NR) Library, and the abundance of the species was calculated using the corresponding abundance of the genes. In order to construct the abundance profile on the corresponding taxonomy level, abundance statistics were performed at each level of Domain, Kingdom, Phylum, Class, Order, Family, Genus and Species.

Linear discriminant analysis Effect Size (LEfSe), is an analytical tool designed to discover and interpret biomarkers in high-dimensional data. It can perform comparisons between two or more groups, emphasizing statistical significance and biological relevance, and can find statistically significant biomarkers between groups. Initially, the non-parametric Kruskal-Walli's rank-sum test is used in multiple sample groups to detect species with significant abundance differences between different groups. Then, the significantly different species obtained from the previous step are used for intergroup difference analysis with the Wilcoxon rank-sum test. Finally, linear discriminant analysis (LDA) is used to reduce the dimensionality of the data and evaluate the influence of the significantly different species.

Integrative analysis

Single-cell identification of subpopulations with bulk sample phenotype correlation (Scissor, v2.0) method was used to identify bulk phenotype-associated cell

subpopulations [11]. In our study, the input data of Scissor pipeline consisted of scRNA-seq data, bulk RNA-Seq expression matrix and matched clinical outcomes. Then, the correlation matrix was constructed to quantify the similarity between the single-cell data and bulk data. Based on the signs of the estimated logistic regression coefficients, the single cells were classified into response and nonresponse cells, which were positively and negatively associated with the matched clinical outcomes, respectively. We set the parameter α equals to 0.05 in the above implementation.

For quantifying metabolism activity, we performed Flux balance analysis to derive 13,082 metabolic fluxes and 142 metabolic pathways for single cell and bulk RNA-seq datasets by METAFlux (v1.0) R package [12].

Data from WMS and metabolomic profiling were integrated using MetOrigin to assign host, microbiome, and co-metabolism activities [13].

References:

1. American PA. American Psychiatric Association: Diagnosti c and Statistical Manual of Mental Disorders, Arlington. 2013.
2. Hao Y, Hao S, Andersen-Nissen E, Mauck WM, Zheng S, Butler A, et al. Integrated analysis of multimodal single-cell data. *CELL*. 2021;184:3573-3587.
3. Ianevski A, Giri AK, Aittokallio T. Fully-automated and ultra-fast cell-type identification using specific marker combinations from single-cell transcriptomic data. *NAT COMMUN*. 2022;13.
4. Wu T, Hu E, Xu S, Chen M, Guo P, Dai Z, et al. clusterProfiler 4.0: A universal enrichment tool for interpreting omics data. *INNOVATION-AMSTERDAM*. 2021;2:100141.
5. Skinnider MA, Squair JW, Kathe C, Anderson MA, Gautier M, Matson KJE, et al. Cell type prioritization in single-cell data. *NAT BIOTECHNOL*. 2021;39:30-34.
6. Jin S, Guerrero-Juarez CF, Zhang L, Chang I, Ramos R, Kuan C, et al. Inference and analysis of cell-cell communication using CellChat. *NAT COMMUN*. 2021;12.
7. Qiu X, Hill A, Packer J, Lin D, Ma YA, Trapnell C. Single-cell mRNA quantification and differential analysis with Census. *NAT METHODS*. 2017;14:309-315.
8. Love MI, Huber W, Anders S. Moderated estimation of fold change and dispersion for RNA-seq data with DESeq2. *GENOME BIOL*. 2014;15:550.
9. Langfelder P, Horvath S. WGCNA: an R package for weighted correlation network analysis.

BMC BIOINFORMATICS. 2008;9.
10. Pang Z, Chong J, Zhou G, de Lima Morais DA, Chang L, Barrette M, et al. MetaboAnalyst 5.0: narrowing the gap between raw spectra and functional insights. *NUCLEIC ACIDS RES*. 2021;49:W388-W396.
11. Sun D, Guan X, Moran AE, Wu L, Qian DZ, Schedin P, et al. Identifying phenotype-associated subpopulations by integrating bulk and single-cell sequencing data. *NAT BIOTECHNOL*. 2022;40:527-538.
12. Huang Y, Mohanty V, Dede M, Tsai K, Daher M, Li L, et al. Characterizing cancer metabolism from bulk and single-cell RNA-seq data using METAFlux. *NAT COMMUN*. 2023;14.
13. Yu G, Xu C, Zhang D, Ju F, Ni Y. MetOrigin: Discriminating the origins of microbial metabolites for integrative analysis of the gut microbiome and metabolome. *iMeta*. 2022;1.
