# Supplementary material for: Single-Cell RNA Transcriptomics and Multi-omics Analyses Reveal the Clinical Effects of Acupuncture on Methadone Reduction
Source: Research (Wash D C). 2025 Jun 24;8:0741. doi: 10.34133/research.0741 (PMC12187353; doi:10.34133/research.0741)
Supplement: Supplementary 1 — Figs. S1 to S6 Tables S1 to S11 Supplementary Methods Trial Protocol [file research.0741.f1.zip › Trial Protocol.pdf]

Supplementary Materials*

Clinical Trial Protocol

Statistical Analysis Plan

* This supplementary material was provided by the authors to give readers further details on their article. The material was not copyedited.

Effect of Manual Acupuncture on Methadone Reduction in Patients undergoing Methadone Maintenance Treatment: Protocol for A Multicenter, Randomized Clinical Trial

Clinical sites:

1. The Affiliated Brain Hospital of Guangzhou Medical University, Guangdong, China.
2. Guangzhou Baiyun District Maternal and Child Health Hospital, Guangdong, China.
3. Shunde Wu Zhong Pei Hospital, Guangdong, China.
4. The Third People's Hospital of Foshan, Guangdong, China.
5. The Third People's Hospital of Zhaoqing, Guangdong, China.
6. Zhongshan Second People's Hospital, Guangdong, China.

Data Management and Statistical Centers:

Clinical Research and Big Data Laboratory, South China Research Center for Acupuncture and Moxibustion, Medical College of Acu-Moxi and Rehabilitation, Guangzhou University of Chinese Medicine

Trial registration

This trial has been registered at Chinese Clinical Trial Registry, with approval number ChiCTR2200058123.

Protocol version

Version number: 3.0. Version data: February 20, 2022

Funding

This study was supported by National Natural Science Foundation of China (82174527); the special project of "Lingnan Modernization of Traditional Chinese Medicine" within the 2019 Guangdong Provincial Research and Development

Program (2020B1111100008); the Innovation Team and Talents Cultivation Program of the National Administration of Traditional Chinese Medicine (ZYYCXTD-C-202004); and the Project of First Class Universities and High-level Dual Discipline for Guangzhou University of Chinese Medicine.

Roles and responsibilities:

The protocol was designed by Liming Lu, Xiaojing Wei, Rouhao Chen. The funder had no role in study design, collection, management, analysis, and interpretation of data; writing of the report; and the decision to submit the report for publication.

Table of Contents

1. Study Contact and Organization ... 5
    1.1 Study Contacts ... 5
    1.2 Recruiting Sites ... 5
2. Introduction ... 6
    2.1 Background ... 6
    2.2 Study Objectives and Hypothesis ... 9
3 Study Design and Methods ... 10
    3.1 Study Design ... 10
    3.2 Randomization, Allocation, and Blinding ... 10
4. Eligibility Criteria ... 11
    4.1 Inclusion Criteria ... 11
    4.2 Exclusion Criteria ... 12
    4.3 Recruitment ... 12
5. Intervention ... 12
    5.1 Manual Acupuncture Group ... 13
    5.2 Sham Control Group ... 15
6. Outcome ... 17
    6.1 Primary Outcomes ... 17
    6.2 Secondary Outcomes ... 17
7. Urine Test ... 19
8. Safety ... 19
9. Participant Timeline ... 20
10. Sample Size Calculation ... 20
11. Statistical Analysis ... 21
12. Research Ethics Approval and Consent or Assent ... 22
13. Quality Control, Data Management and Monitoring ... 23
14. Dissemination Policy ... 23
Abbreviations ... 23
References ... 24
Appendix 1: Diagnostic criteria for opioid use disorder (DSM-5) ... 36
Appendix 2: General principles for dosing determination of methadone maintenance treatment .. 37
Appendix 3: Opioid Craving VAS ... 38
Appendix 4: Clinical Opiate Withdrawal Scale (COWS) ... 39
Appendix 5: Pittsburgh Sleep Quality Index (PSQI) ... 41
Appendix 6: The Beck Anxiety Inventory (BAI) ... 43
Appendix 7: Beck Depression Inventory-II (BDI-II) ... 44
Statistical Analysis Plan ... 48

1. Study Contact and Organization

1.1 Study Contacts

Principal Investigator for Study
Liming Lu, PhD

Clinical Research and Big Data Laboratory, South China Research Center for Acupuncture and Moxibustion, Medical College of Acu-Moxi and Rehabilitation, Guangzhou University of Chinese Medicine, Guangzhou, 510006, P.R. China;

Email: lulimingleon@126.com

1.2 Recruiting Sites

Shichao Xu

Affiliated Brain Hospital of Guangzhou Medical University (Guangzhou Huiai Hospital), 510370, P.R. China;

Email: xscmate@139.com

Fang Cao

Guangzhou Baiyun District Maternal and Child Health Hospital, 510006, P.R. China.

Email: addaaddaaaaa1@sina.com

Hong Gu

Shunde Wu Zhong Pei Hospital, 528300, P.R. China

Email: wuzhongpeiywk@126.com

Yiliang Liu

The Third People's Hospital of Foshan, 528000, P.R. China

Email: pnslyl@163.com

Zhiqiu He

The Third People's Hospital of Zhaoqing, 526000, P.R. China

Email: 1080904490@qq.com

Guodong Mo
Zhongshan Second People's Hospital, 528400, P.R. China
Email: moguodong2002@163.com

2. Introduction

2.1 Background

2.1.1 The situation of opiate abuse remains grave

Opioid Use Disorder (OUD) represents an addictive condition characterized by intense cravings for opiate substances, increased tolerance, difficulty in reducing usage, and notable withdrawal symptoms upon cessation [1,2]. Opioid substances encompass both natural and synthetic compounds. The former includes poppy, opium, morphine, and heroin, while the latter mainly consists of methadone, pentazocine, buprenorphine, and other medically synthesized opioid analgesics like fentanyl [3]. Both domestic and international scenarios regarding opiate substance abuse face daunting challenges. According to the "World Drug Report 2021" [4], as of 2020, approximately 275 million individuals globally were drug users, with opiate drugs still contributing the most to the disease burden caused by illicit substances. Moreover, over the past decade, the global usage of opioid drugs has nearly doubled. In 2019 alone, drug abuse resulted in almost 500,000 deaths worldwide, with 70% attributed to the misuse of opioid drugs. By the end of 2020, around 1.8 million people were using illicit drugs in China, with 734,000 opiate users accounting for 40.8% of the total drug users [5]. Opioid dependence poses significant threats and risks to public health and safety.

2.1.2. A large population undergoing methadone maintenance treatment (MMT) has a demand for methadone dose reduction

Methadone, an artificially synthesized opioid medication, is the cornerstone of

methadone maintenance treatment, a community-based substitution therapy for opioid substances. MMT uses legal methadone to replace of illegal opioid to treat opioid substance dependence [6,7]. It is the main intervention for the treatment of OUD worldwide [8,9]. The effectiveness of MMT in treating OUD is unquestionable, but associated issues have become increasingly apparent. As a synthetic opioid medication, methadone carries addictive potential and the risk of misuse [10]. Its toxicity exposes patients to a heightened risk of QT prolongation and arrhythmias, even at lower doses [11,12]. As the duration of treatment progresses, tolerance to methadone also increases, necessitating higher doses to maintain therapeutic effects, thereby raising the risk of overdose-related fatalities [13]. Moreover, reports indicate a persistent rise in constipation among MMT clients [14], with insomnia detection rates ranging from 58.2% to 85.8% [15]. Patients experience varying degrees of anxiety, depression, and other symptoms for a considerable period [16], while MMT-related sexual dysfunction is also prevalent [17-19]. Furthermore, the longer the duration of methadone treatment, the higher the likelihood of heroin misuse behavior [20].

MMT clients have a need to reduce the methadone dose, but the effectiveness of reducing the methadone dose by themselves is limited [21,22]. Due to the impact of side effects and aspirations for complete recovery, patients gradually reduce their doses to achieve complete cessation of medication and detoxification. Studies have shown that 40% to 57% of patients undergo the dose reduction process [22-26], and following adequate maintenance treatment, patients can gradually taper off with lower doses and attempt medication cessation [27]. Therefore, MMT clients necessitate effective and safe treatment methods to achieve methadone dose reduction, alleviate methadone toxicity, and concurrently reduce relapse behavior triggered by drug cravings.

2.1.3 Acupuncture has the potential to ameliorate withdrawal symptoms and alleviate drug cravings associated with opioid medications. There is still a lack of robust evidence in this field.

Acupuncture, as an integral component of traditional Chinese medicine, boasts a

history spanning thousands of years in disease treatment. It is characterized by its simplicity of practice and remarkable therapeutic efficacy, which has led to its endorsement by the World Health Organization (WHO) for treating over 40 different conditions [28]. Also, acupuncture has been recognized by the WHO as a non-pharmacological therapy for treating drug dependence, with the potential to reduce relapse by inhibiting attentional bias towards heroin, mitigating complications associated with drug dependence and facilitating opiate withdrawal syndrome [29-32].

Several studies have identified the potential role of acupuncture in improving opioid dependence by reducing opioid use and diminishing craving. [33-35]. In a randomized controlled trial (RCT) conducted by Chan et al. [36] indicated that compared to the sham group, the real four-week-acupuncture group exhibited a significant reduction in daily methadone consumption and a notable improvement in sleep latency. In a pilot RCT by Deng et al. [37], acupuncture was found to assist in reducing medication dosages among patients already using opioids and alleviate opioid cravings among individuals not currently on opioid medication. Another study by Crawford et al. [38] discovered a 45% reduction in the overall prescription of opioids among 172 patients who had undergone acupuncture treatment at a United States Air Force medical center at least four times. Baker et al. [39] conducted a systematic review suggesting that auricular acupuncture may be an effective adjunctive therapy, improving methadone retention rates and reducing the maintenance dose.

Our group has previously conducted a clinical trial and literature review to initially explore the potential of acupuncture in reducing the daily consumption of methadone and opioid cravings in MMT clients. Our RCT demonstrated that acupuncture surpasses standalone methadone therapy in reducing methadone dosage, improving drug cravings, and addressing insomnia without concomitantly increasing the rate of positive urine tests for opioids [40]. Meanwhile we also assessed the economic value of acupuncture from a health economics perspective in this trial [41]. Another study conducted a comprehensive and systematic evaluation of acupuncture and non-pharmacological therapies for improving drug dependence and addressing

neurological symptoms, revealing the potential of acupuncture to alleviate withdrawal symptoms and enhance neurological function among individuals with substance dependence [42,43].

In general, although there have been several studies focusing on acupuncture as an adjunct treatment for drug addiction, they have predominantly consisted of small-sample clinical trials without the inclusion of sham acupuncture as a control. Consequently, there is a compelling need to undertake high-quality prospective RCT incorporating sham acupuncture as a control in order to substantiate and affirm the efficacy and safety of acupuncture in MMT, thereby providing more persuasive evidence.

2.1.4 Summary

As of the end of 2019, there were 764 community MMT clinics treating approximately 112,000 individuals in China [7]. The large scale of the MMT population has significantly increased healthcare costs and consumed social workforce and welfare resources, imposing a heavy burden on patients, their families, healthcare providers and society. The intervention efforts targeting the MMT population hold great importance in promoting public health and well-being in our country. Therefore, based on previous studies, this study establishes a placebo acupuncture group as a control and executes a more comprehensive and rigorous RCT to further validate the efficacy of acupuncture in reducing methadone dose and preventing relapse among MMT clients. The objective is to provide effective treatment strategies as a reference for MMT clients to reduce their methadone reliance and alleviate the burdensome side effects while offering high-quality clinical evidence for the use of acupuncture for MMT clients.

2.2 Study Objectives and Hypothesis

The aim of this study is to evaluate the efficacy and safety of manual acupuncture intervention for MMT clients through a multicenter clinical randomized controlled trial, comparing it with non-penetrating sham acupuncture.

3 Study Design and Methods

3.1 Study Design

This is a parallel-arm, patient-blinded, multicenter, randomized, controlled trial. All eligible individuals will be randomly assigned to the manual acupuncture group or the sham control group through central randomization in a 1: 1 ratio. The time period of this trial consists of one week of baseline assessment, 8 weeks treatment period following randomization and a follow-up period of an additional 12 weeks post-treatment. The study flowchart of study procedure is presented in **Figure 1**.

3.2 Randomization, Allocation, and Blinding

Eligible patients are randomly assigned in an equal ratio to the manual acupuncture group or the sham control group via a central randomization system designed by the Clinical Research and Big Data Laboratory, South China Research Center for Acupuncture and Moxibustion, Guangzhou University of Chinese Medicine (http://47.119.117.255/). The randomization sequence is generated in the block size of 4. Participants will be informed that they have an equal chance to be assigned to the manual acupuncture group or the sham control group. After participants sign the informed consent form and complete baseline assessments, the researcher applies for the randomization number and allocates participants to each group. Allocation of participants is performed by an independent researcher at the clinical site who is not involved in the outcome assessment. Due to the nature of this study, acupuncturists are not blinded to group allocation. However, the outcome assessors, physicians who prescribe patient's methadone, national government personnel who is responsible for recording daily consumption of methadone, data collectors, and statisticians are all blinded to group allocations. Besides, an independent researcher supervises the conversation between acupuncturists and physicians to avoid them communicating issues related to the allocation. Upon the culmination of 8 weeks intervention, patients will be asked to guess whether they perceived the insertion of the needle tip into their skin to assess the efficacy of blinding maintenance.

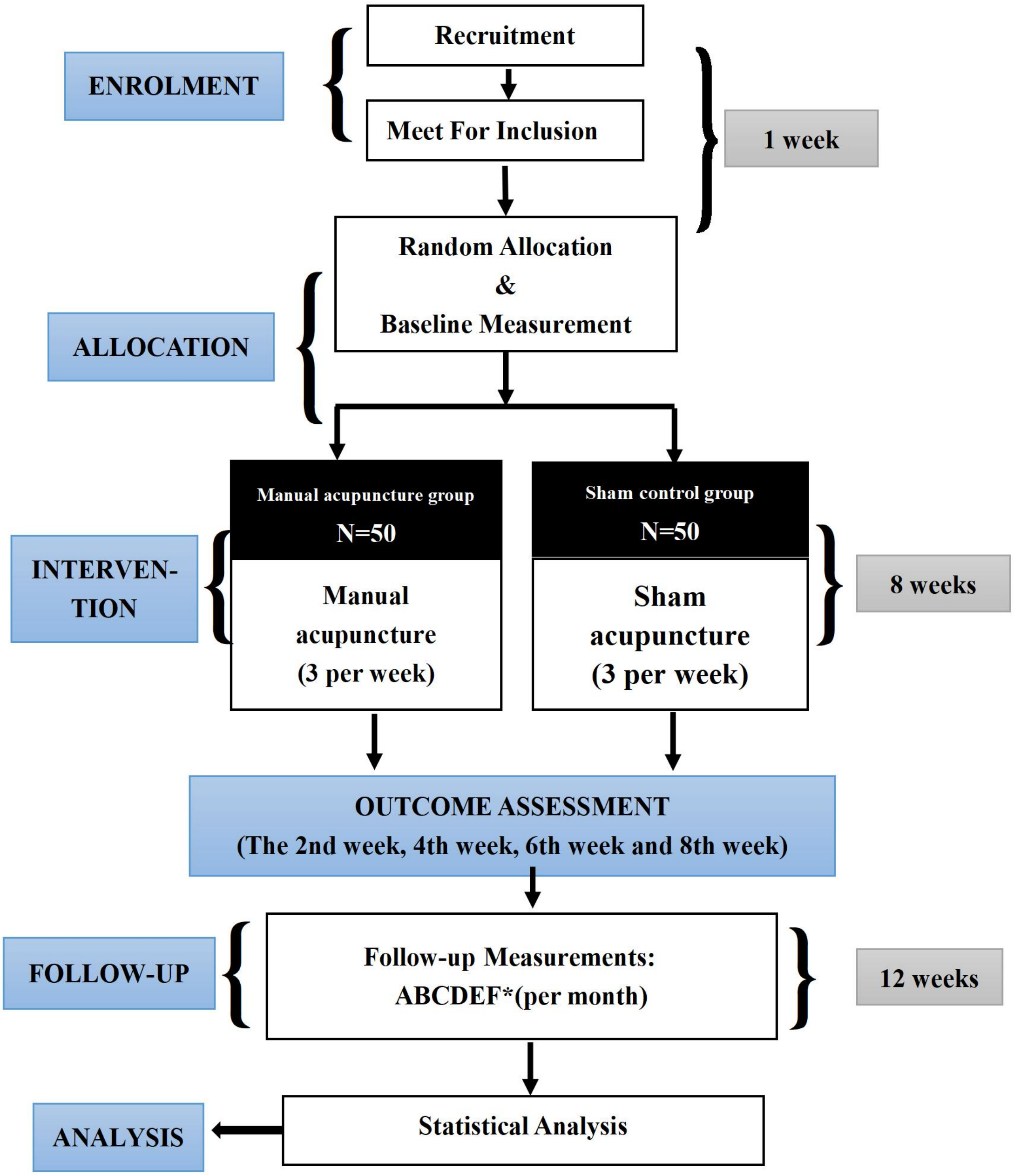

Figure 1: Flowchart of trial procedures

*A: Daily Consumption of Methadone; B: Visual Analog Scale (VAS) for opioid craving; C: Clinical Opioid Withdrawal Scale (COWS); D: Beck Anxiety Inventory (BAI); E: Beck Depression Inventory II (BDI-II); F: Pittsburgh sleep quality index (PSQI)

4. Eligibility Criteria

4.1 Inclusion Criteria

Patients will be included if they have:

- Diagnosed as opioid dependence according to the fifth edition of the Diagnostic and Statistical Manual of Mental Disorders (DSM-5) (Appendix 1) [44];
- Aged between18-65 years;
- Received MMT for more than 6 weeks;
- Demand for methadone tapering;
- Not having received any kind of acupuncture therapy during the previous 3 months.

4.2 Exclusion Criteria

Patients will be excluded if they have:

- Serious heart, liver, lung, or kidney diseases;
- Syphilis or AIDS;
- Severe digestive disease or malnutrition;
- Severe primary hematological disorders;
- Received other treatment that may affect the efficacy evaluation of the present intervention;
- Infection, inflammation, scar, or injury close to the site of the selected acupoints;
- Pregnancy, or plans for pregnancy;
- Had a history of mental illness other than drug dependence.

In addition, all patients are instructed not to take any other medications aiming at alleviating withdrawal symptoms, improving sleep quality, or managing depression and anxiety during the study, in order to avoid initiating other interventions. Patients who consume such medications are excluded from the study.

4.3 Recruitment

All patients will be recruited in the outpatient clinics of the participating hospitals by local advertising, hospital website, and hospital WeChat Public Accounts.

5. Intervention

Acupuncture treatment will be performed by licensed acupuncturists who has at least 3 years of acupuncture experience. All participants will receive 24 sessions of

30-minutes acupuncture over 8 weeks. We set up the standardized ritual operations and perform them in both manual acupuncture and sham control group.

5.1 Manual Acupuncture Group

Manual acupuncture will be applied at acupoints that belong to Jin's three-needle acupuncture (JTN). JTN is a clinical acupuncture school which has been successfully applied in several RCTs [45,46]. This school always combines three acupoints that have a synergistic effect as an acupoints group. Among them, we will choose "Dingshen-zhen", "Sishen-zhen" and "Shouzhi-zhen" which are commonly used to treat psychiatric disorders such as opiate withdrawal [47]. The location of these points is described in **Table 1** and **Figure 2**.

Sterile stainless-steel disposable acupuncture needles (Huatuo, Suzhou, China; lengths and diameters: 25 mm × 0.3 mm) will be used in this group. Among the chosen points, Shouzhi-zhen will be needled at an angle of 45-90° to the participant's skin, while Dingshen-zhen and Sishen-zhen will be needled at an angle of 15-30° to the skin. The needles will be inserted at a depth of 5-30 mm. Then, the needles will be manually stimulated to achieve the typical acupuncture sensation of "de qi" which is characterized as soreness, numbness, and heaviness. Over a 30-minute period, manual manipulation for each acupoint lasts 10 seconds and is repeated three times with intervals of 10 minutes.

Table 1 Acupoints used in the manual acupuncture group

Acupoints	Location	Depth of insertion
Sishen-I (GV21: Qianding)	On the head, 3.5B-cun superior to the anterior hairline, on the anterior median line.	0.5-0.8 cun
Sishen-II (GV19: Houding)	On the head, 5.5B-cun superior to the posterior hairline, on the posterior median line.	0.5-0.8 cun
Sishen-III	On the head, 1.5 cun left lateral to the anterior median line and at the same level as GV20. GV20 is on the head, 5B-cun superior to the anterior hairline, on the anterior median line.	0.5-0.8 cun
Sishen-IV	On the head, 1.5 cun right lateral to the anterior median line and at the same level as GV20.	0.5-0.8 cun
Dingshen-I	On the head, directly 0.5 cun superior to EX-HN3. EX-HN3 is on the head, between the right medial end of the eyebrow and the left one.	0.5-0.8 cun
Dingshen-II	On the head, directly 0.5 cun superior to left GB14. GB14 is on the head, 1B-cun superior to the eyebrow, directly superior to the centre of the pupil.	0.5-0.8 cun
Dingshen-III	On the head, directly 0.5 cun superior to right GB14	0.5-0.8 cun
Shouzhi-I *(bilateral)* (HT7: Shenmen)	On the anteromedial aspect of the wrist, radial to the flexor carpi ulnaris tendon, on the palmar wrist crease.	0.5-0.8 cun
Shouzhi-II *(bilateral)* (PC6: Neiguan)	On the anterior aspect of the forearm, between the tendons of the palmaris longus and the flexor carpi radialis, 2B-cun proximal to the palmar wrist crease.	0.8-1.0 cun
Shouzhi-III *(bilateral)* (PC8: Laogong)	On the palm of the hand, in the depression between the second and third metacarpal bones, proximal to the metacarpophalangeal joints.	0.5-0.8 cun

Note: The prescribed acupoints come from the WHO Standard Acupuncture Point Locations in the Western Pacific Region. The *"Shouzhi-I", "Shouzhi-II", "Shouzhi-III"* only needles one hand each time, and both sides take turns for treatment.

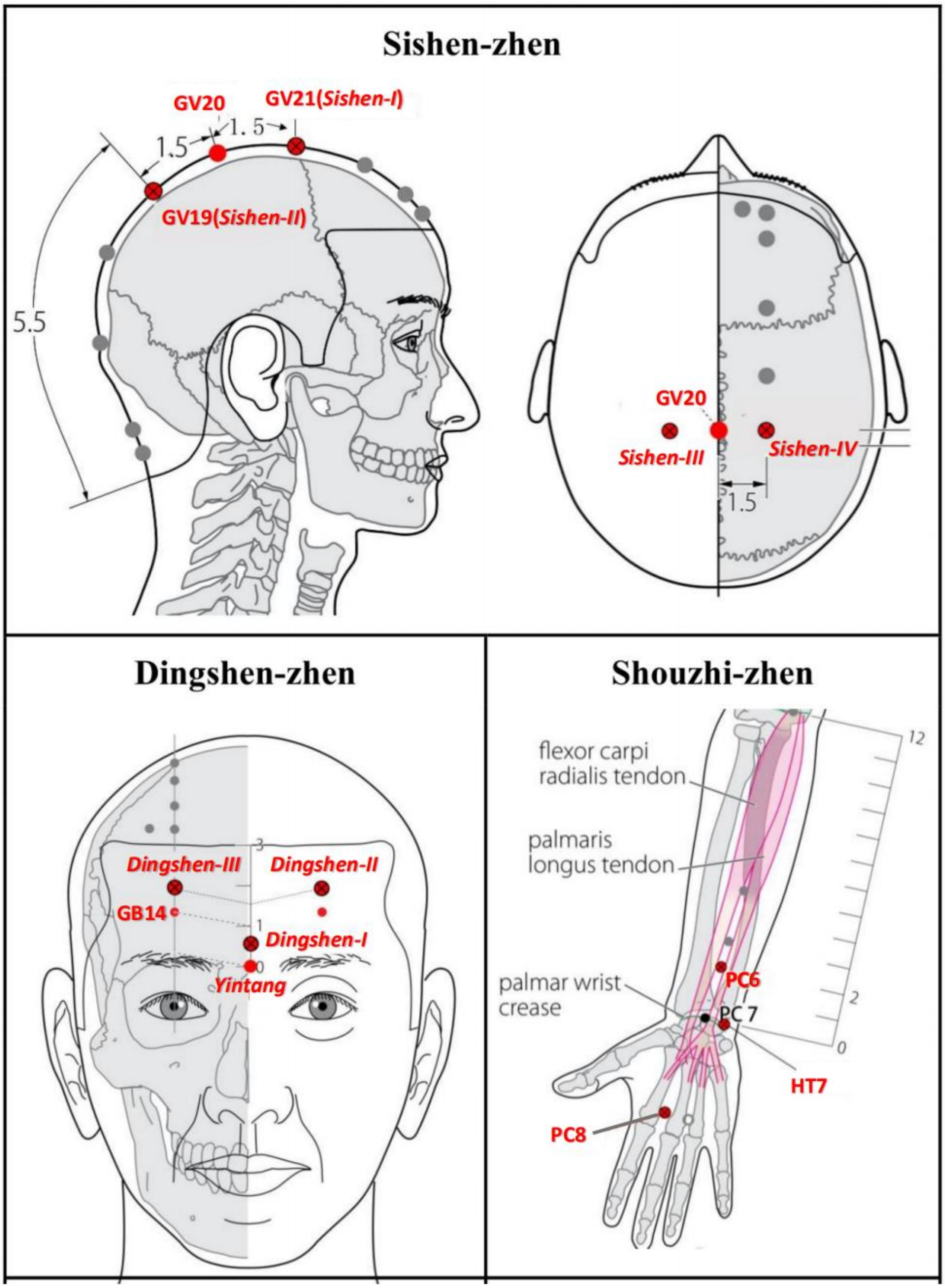

Figure 2 Acupoints used in the manual acupuncture group (The lower right corner shows only the right hand acupoints)

5.2 Sham Control Group

Non-penetrating sham acupuncture will be applied at the same acupoints as the

manual acupuncture group. Sterile stainless-steel disposable blunt acupuncture needles (Huatuo, Suzhou, China; lengths and diameters: 25 mm × 0.3 mm) are used as sham needles. When they are fixed on the skin through acupuncture auxiliary device, patients fell a pricking sensation, simulating a puncture of the skin. However, the needles do not penetrate the skin when they are pressed by acupuncturists.

Patient-blinded acupuncture intervention uses a special acupuncture auxiliary device (**Figure 3**). The device has already been granted a patent by China's national intellectual property administration (No. ZL 202223328917.3). The device consists of a plastic pedestal and a guide tube, and the shape of the device used is the same in both groups. For real acupuncture, we use hollow instruments where needles can be inserted hygienically directly into the skin. For placebo acupuncture, we use non-hollow instruments and blunt needles. To achieve a single-blind outcome, the acupuncturist fixes an acupuncture auxiliary and a needle or placebo needle to the skin of the acupoint after sterilization. Then the acupuncturist quickly taps the top of the tube to make the needle go downward and removes the tube. Therefore, participants are blinded to group and intervention by entering the needle through this acupuncture auxiliary device.

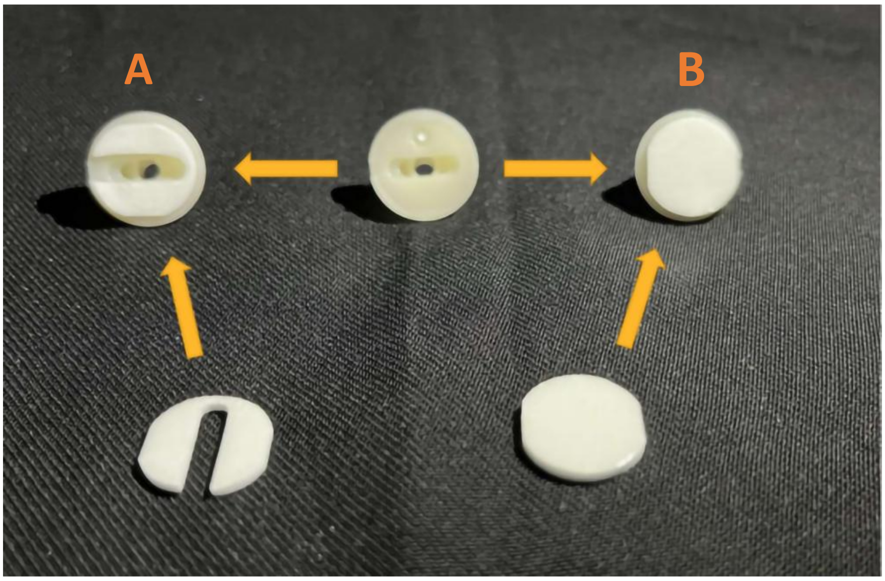

Figure 3 acupuncture auxiliary device (A: hollow instruments for real acupuncture. B: non-hollow instruments for placebo acupuncture)

6. Outcome

6.1 Primary Outcomes

6.1.1 The Rate of Methadone Dose Reduction

One primary outcome is the rate of methadone dose reduction, defined as the proportion of participants who achieve a reduction in methadone dose of ≥20% compared to baseline after 8 weeks of intervention. Based on the results of previous studies and the standards and clinical guidelines of MMT in the United States and Canada, we find that achieving a reduction in methadone dose of ≥20% at the end of intervention is associated with significant clinical benefits[40,41,48,49]. Therefore, a 20% reduction in methadone dose is used as the threshold to define significant improvement in tapering effectiveness.

Enrolled patients come to visit the clinic every day to take methadone, which is strictly administered by the physicians as required by the MMT clinics. Daily consumption of methadone from baseline to the 20th week will be obtained from physicians and recorded by independent research assistants, which is used to calculate average daily methadone dose per week and the rate of methadone dose reduction.

6.1.2 Opioids Craving Visual Analog Scale

The other primary outcome is the opioid craving using a visual analog scale for drug craving marking on a 100-millimetre line in proportion to their craving from 0 (no craving) to 100 (strong craving)[50]. The degree of craving will be marked by subjects at baseline, the 2nd, the 4th, the 6th, and the 8th week after acupuncture administration and at the post-treatment follow-up visits in the 12th, the 16th, and the 20th week. The minimal clinically important difference (MCID) for the opioid craving VAS is 4.0 points [51,52].

6.2 Secondary Outcomes

6.2.1 Clinical Opioid Withdrawal Scale (COWS)

Opioid withdrawal symptoms will be assessed using the Chinese version of the

COWS [53] from baseline to the 2nd, the 4th, the 6th, and the 8th week after acupuncture administration and at post-treatment follow-up visits in the 12th, the 16th, and the 20th week. COWS evaluates common signs and symptoms of opioid withdrawal and monitors these symptoms over time. The scale consists of 11 items, including heart rate, gastrointestinal discomfort, sweating, tremor, yawning and other symptoms related to opioid withdrawal. Each item has 4 options, and the mark can best describe the root symptoms or signs of the subject option. The total score of 11 items is calculated: 5-12 scores represent mild, 13-24 moderate, 25-36 serious, and above 36 severe withdrawal symptoms.

6.2.2 Pittsburgh Sleep Quality Index (PSQI)

Sleep quality will be assessed using the Chinese version of the PSQI[54] from baseline to the 4th, the 8th, the 12th, the 16th and the 20th week. The Chinese version of PSQI has demonstrated reliability and validity similar to that of the original language version [55]. PSQI evaluates sleep disturbances through subjective sleep quality, sleep duration, sleep latency, habitual sleep efficiency, sleep disturbances, daytime dysfunction and use of sleeping medication. Each item is graded on 4-point (0–3), which is summed together and gains a total score (0 to 21). Higher scores show that patients suffer severity of sleep disturbance and a total score higher than 5 indicates “poor sleep”.

6.2.3 The Beck Depression Inventory-II (BDI-II)

BDI-II, a self-reported questionnaire, will be used to assess the severity of depression, which consists of 21 symptoms [56]. Each symptom is graded on 4-point (0–3), which is summed together to yield a total score (0 to 63). Higher scores mean greater severity of depression. The Chinese version has been validated in adolescents[57]. The BDI-II will be assessed at baseline, the 4th, the 8th, the 12th, the 16th, and the 20th week.

6.2.4 The Beck Anxiety Inventory (BAI)

Anxiety symptoms will be assessed by the BAI, which is a questionnaire with 21

items [58]. Each symptoms rate is on a 4-point (0-3). The maximum score is 63 and higher scores indicate greater severity of anxiety. The BAI will be assessed at baseline, the 4th, the 8th, the 12th, the 16th, and the 20th week.

Independent research assistants will supervise patients to complete the opioid craving VAS and all secondary outcome measures in pen and paper format and evaluated their outcomes.

7. Urine Test

All participants will be asked to submit a urine sample at baseline, and randomly during the study each month, which are used to test for opioid use.

8. Safety

All participants will have some tests to check up on their bodies at screening and after 8-week intervention. Those tests are relative to the heart, liver, kidney, and other organs, including white blood cell count, hematocrit, hemoglobin, platelet, aspartate aminotransferase/alanine aminotransferase, blood urea nitrogen, creatinine, gamma-glutamyl transpeptidase, erythrocyte sedimentation rate and electrocardiogram.

Adverse events (AEs) are defined as events associated with acupuncture including fainting, hematoma, broken needle, infection, and bleeding. If any adverse events occur, these will be recorded by the acupuncturist and the treatment will be temporarily stopped. Every AEs will be fully recorded on the case report forms (CRFs) including all details of AEs. Serious AEs that threaten to participant's life or result in hospitalization will be reported to the Research Ethics Committee concerned within 24 hours.

9. Participant Timeline

The participant timeline is described in **Figure 4**.

	STUDY PERIOD												
	Enrolment	Allocation	Treatment phase								Follow-up		
**Timepoint (week)**	−1	0	1	2	3	4	5	6	7	8	12	16	20
**ENROLLMENT:**	×												
Eligibility screen	×												
Informed consent	×												
Allocation		×											
**INTERVENTIONS:**													
Manual Acupuncture			◆	—	—	—	—	—	—	▶			
Sham Acupuncture			◆	—	—	—	—	—	—	▶			
**ASSESSMENTS:**													
Daily Consumption of Methadone		×		×		×		×		×	×	×	×
opioid craving VAS		×		×		×		×		×	×	×	×
COWS		×		×		×		×		×	×	×	×
BAI		×				×				×	×	×	×
BDI-II		×				×				×	×	×	×
PSQI		×				×				×	×	×	×
**ADVERSE EVENTS**:			×	×	×	×	×	×	×	×	×	×	×

Figure 4: Schedule of enrolment, interventions, and assessments

10. Sample Size Calculation

Estimates of clinically meaningful differences between the manual acupuncture group and the sham control group are based on the data of Nosyk et al. and our previous study [18,19,34]. We assume an efficacy rate of 70% for the manual acupuncture

group and 36% for the sham control group, 42 participants per group are needed at 5% significance with 90% power. This provided exceeding 99% power to detect a mean difference of 10 (SD 8) in opioid craving VAS scores between the two groups at a 5% significance level for a 2-sided test (required 25 participants per group). Thus, anticipating approximately 15% attrition, at least 50 participants per group (n = 100 in total) are required.

11. Statistical Analysis

Statistical analysis will be performed by the Clinical Research and Big Data Laboratory, South China Research Center for Acupuncture and Moxibustion, Guangzhou University of Chinese Medicine. The statistician will be blinded to group allocation until final unblinding. SAS 9.4 will be utilized for data analysis. Missing data are filled by using multiple imputation.

Continuous variables are reported as the mean with standard deviation or median and interquartile range. Categorical variables are described as numbers and percentages using $\chi 2$ test or Fisher's exact test. Longitudinal measures through 20 weeks (8 weeks of intervention and 12 weeks of follow-up) are analyzed for all outcomes. The analysis of proportion of participants with at least 20% reduction from baseline in the amount of methadone dose is performed using a generalized linear mixed-model. For opioid craving VAS and all secondary outcomes, analysis is conducted utilizing a linear mixed-model. For above models, values measured at baseline are used as covariates; intervention assignment, visit and intervention × visit interaction are as fixed effects; and treatment centre and centre × intervention interaction are as random effects. We assessed the poolability of treatment effects across centres by including centre × intervention interaction in the model. If P-values corresponding to the centre × intervention interaction term for both primary endpoints are greater than 0.10, the treatment effect is homogenous across centres [59].

Dynamic data will be demonstrated on the reduction in the methadone consumption and the opioid craving VAS score in the figures, separated by the manual acupuncture and sham group. Each row in the figures represents the reduction dose of

the daily consumption of methadone (the opioid craving VAS score) for one participant. Individuals who discontinue participation early appear at the bottom of each plot, whereas those with complete data appear at the top.

To investigate the relationship between daily consumption of methadone and the intensity of opioid craving over a 20-week period, scatter plots are employed as a visual representation in this study with the x-axis representing the opioid craving VAS and the y-axis representing the daily consumption of methadone. The resulting scatter plot allows for the examination of the correlation between these two variables, providing insights into the potential influence of methadone intake on opioid craving levels over time.

Efficacy analyses will be performed for both the full analysis set (FAS) population and the per-protocol set (PPs) population. The FAS population will consist of all randomized patients who received at least one treatment. In a PPs analysis, only patients who complete all the clinical trials according to the protocol are counted towards the results. Besides, the PPs population in our research will meet these criteria: the experimental treatment requirements; the main variables can be determined; no missing baseline variables; no major violation of the test scheme. Safety analyses will be performed on the safety population, which will be comprised of all randomized subjects who have been administered at least one treatment. A PP analysis is carried out as a sensitivity analysis compared with the results obtained using the FAS analysis. When the PPs and FAS analysis conclusions are not the same, it is necessary to analyze the possible reasons and discussion them.

Primary outcomes used 2-sided tests at the 2.5% significance level. All other statistical tests are 2 -sided at the 5% significance level.

12. Research Ethics Approval and Consent or Assent

The trial is designed in accordance with the principles of the Declaration of Helsinki. The trial protocol (version number: 3.0, version date: February 20, 2022) has been approved by the Ethics Committee of the Panyu Hospital of Traditional Chinese Medicine (Ethics approval numbers: 2022029) on February 22, 2022. Written

informed consent will be obtained from each participant before they enter the trial.

13. Quality Control, Data Management and Monitoring

Before recruitment, the entire team will be required to attend a training workshop, which ensures strictly comply with the study protocol and totally understanding of the trial administration process. The interventions will be performed by licensed acupuncturists with more than three years of experience in hospitals or clinics. They are also received a brochure about the protocol and standard operation procedure for acupuncture.

The data will be carefully collected and recorded on CRFs. All data will be put onto the computer with password protection by staff blinded to group allocation and checked twice by investigators after data entry. Data quality will be checked regularly by research assistants and supervised by monitors. The original CRFs and all other forms will be archived securely at the Medical College of Acu-Moxi and Rehabilitation of Guangzhou University of Chinese Medicine. Confidentiality is ensured by storing data in a password-protected database, for which only the research team has the password. Paper-based record forms are identified by numbers and stored in a locked cupboard. Any published patient data are not allowed personal identification, only group data could be published.

14. Dissemination Policy

The results of this trial are expected to be published in peer-reviewed journals and presented at national and international meetings.

Abbreviations

AEs: Adverse events; BAI: Beck Anxiety Inventory; BDI- II: Beck Depression Inventory II; FAS: Full analysis set; JTN: Jin's three-needle acupuncture; MCID: Minimal clinically important difference; MMT: Methadone maintenance therapy, OUD: Opioid Use Disorder; PPs: Per-protocol set; PSQI: Pittsburgh Sleep Quality

Index; RCT: Randomized controlled trial; VAS: Visual analogue scale; WHO: World Health Organization.

References

[1] Reimer J, Vogelmann T, Trümper D, Scherbaum N. Opioid use disorder in Germany: healthcare costs of patients in opioid maintenance treatment. Subst Abuse Treat Prev Policy. (2019) 14:57.

[2] Yang F, Hao W. Application prospect of compound buprenorphine in treating opioid use disorders in China. Chin J Drug Depend. (2020) 29:169–75.

[3] Li Peikai, Zhang Cunmin, Li Jianhua. Review of Opioid Dependence Treatment in China[J]. Chinese Journal of Drug Dependence, 2007(03):162-168

[4] United Nations. World Drug Report 2021 (United Nations publication, Sales No. E.21.XI.8).

[5] Report on Drug Situation in China in 2020 [N]. Report on Drug Control in China, 2011-07-23(003)

[6] Sullivan SG, Wu Z, Rou K, Pang L, Luo W, Wang C, Cao X, Yin W, Liu E, Mi G; National Methadone Maintenance Treatment Working Group. Who uses methadone services in China? Monitoring the world's largest methadone programme. Addiction. 2015 Jan;110 Suppl 1:29-39.

[7] Liu Xuejiao, Luo Wei, Zhang Bo, Wen Pinyuan, Wu Zunyou. Constructing a Risk Prediction Model for Continued Drug Use in Methadone Maintenance Treatment Outpatients Based on Lasso-Logistic Regression [J]. Chinese Journal of Disease Control, 2021, 25(12): 1369-1373+1402.

[8] Mattick RP, Breen C, Kimber J, Davoli M. Methadone maintenance therapy versus no opioid replacement therapy for opioid dependence. Cochrane Database Syst Rev. (2009) 2009:CD002209.

[9] Somogyi AA, Barratt DT, Ali RL, Coller JK. Pharmacogenomics of methadone maintenance treatment. Pharmacogenomics. (2014) 15:1007–27.

[10] Lynch ME. A review of the use of methadone for the treatment of chronic noncancer pain [Internet]. Vol. 10, Pain Research and Management. Hindawi

Limited; 2005 [cited 2020 Sep 14]. p. 133–44. Available from: https://pubmed.ncbi.nlm.nih.gov/16175249/

[11] Justo D, Gal-Oz A, Paran Y, Goldin Y, Zeltser D. Methadone-associated Torsades de Pointes (polymorphic ventricular tachycardia) in opioid-dependent patients [Internet]. Vol. 101, Addiction. Addiction; 2006 [cited 2020 Sep 17]. p. 1333–8. Available from:https://pubmed.ncbi.nlm.nih.gov/16911733/.

[12] Behzadi M, Joukar S, Beik A. Opioids and Cardiac Arrhythmia: A Literature Review. Med Princ Pract. 2018;27(5):401-414.

[13] Deborah Brauser.Top Ten Drugs Tied to Overdose Deaths.[OL].December 12, 2018.Top Ten Drugs Tied to Overdose Deaths (medscape.com)

[14] Hu Yuanyuan. Incidence of constipation in heroin addicts during methadone maintenance treatment [J]. Electronic Journal of Clinical Medicine Literature, 2020,7(31):68.

[15] Li Yi, Liu Xuebing, Zhang Yao. Randomized controlled study on acupuncture improving sleep quality of outpatients treated with methadone maintenance [J]. Chinese Journal of Integrated Traditional Chinese and Western Medicine, 2012,32(08):1056-1059.

[16] Shi Fangzhi, Wei Kecheng, Xu Dongbiao, Li Hua, Li Xiliang, Zhao Yi, Teng Yongsheng, Song Xiaoge. Acupuncture and moxibustion improves sleep disorders and anxiety in 35 cases of heroin addicts [J]. Journal of Anhui University of Traditional Chinese Medicine, 2011, 30(02):37-39.

[17] Wang Peng, Zhu Junhong, Yan Yan, Wang Chunhong, Xu Hanming. A study on the status of sexual function in male methadone maintenance patients [J]. Chinese Sexology, 2007(11): 19-21+36.

[18] Yang Liang, Liao Luqiao. Impairment of sexual function in heroin addicts and its clinical significance [J]. Chinese Sexology, 2005(06): 3-5+11.

[19] Meng Donghua. Research progress on sexual dysfunction and arrhythmia caused by methadone maintenance therapy [J]. Applied Preventive Medicine, 2016,22(02):190-192.

[20] Liao Rui, Gong Yi, Han Jiayu, Yu Bin, Dong Peijie, Yang Shifan, Yang Shujuan. Status quo of methadone maintenance treatment of heroin addicts and analysis of influencing factors of stealing behavior [J]. Modern Preventive Medicine, 2020, 47(15) :2789-2792+2861.

[21] Calsyn DA, Malcy JA, Saxon AJ. Slow tapering from methadone maintenance in a program encouraging indefinite maintenance. J Subst Abuse Treat. 2006 Mar;30(2):159-63.

[22] Nosyk B, Sun H, Evans E, Marsh DC, Anglin MD, Hser YI, Anis AH. Defining dosing pattern characteristics of successful tapers following methadone maintenance treatment: results from a population-based retrospective cohort study. Addiction. 2012 Sep;107(9):1621-9.

[23] Lu Q, Zou X, Liu Y, Gong C, Ling L. Dose Tapering Strategy for Heroin Abstinence among Methadone Maintenance Treatment Participants: Evidence from A Retrospective Study in Guangdong, China. Int J Environ Res Public Health. 2019 Aug 6;16(15):2800.

[24] Center for Substance Abuse, Treatment. Samhsa/Csat Treatment Improvement Protocols. In Medication-Assisted Treatment for Opioid Addiction in Opioid Treatment Programs; Substance Abuse and Mental Health Services Administration: Rockville, MD, USA, 2005.

[25] Nosyk, B.; Marsh, D.C.; Sun, H.; Schechter, M.T.; Anis, A.H. Trends in Methadone Maintenance Treatment Participation, Retention, and Compliance to Dosing Guidelines in British Columbia, Canada: 1996–2006. J. Subst. Abuse Treat. 2010, 39, 22–31.

[26] Chen, J.; Chen, W. Dosage Using in Community Methadone Maintenance Treatment at Clinics in Guangdong and Its Adjustment Strategy. Modern Prev. Med. 2010, 37, 1687–1689.

[27] Zhou Zheng. Study on dose reduction of low-dose methadone maintenance therapy for different populations [J]. Chinese Journal of Drug Abuse Prevention, 2013, 19(04): 206-208+211.

[28] Han J.S. Acupuncture analgesia: areas of consensus and controversy. Pain 2011; 152: pp. S41-S48.

[29] Han J.S. Acupuncture analgesia: areas of consensus and controversy. Pain 2011; 152: pp. S41-S48.

[30] Culliton PD, Kiresuk TJ. Overview of substance abuse acupuncture treatment research. J Altern Complement Med. (1996) 2:149–59, 161–5.

[31] Jiang YP, Liu H, Xu P, Wang Y, Lu G-H. Effffect of electro-acupuncture intervention on cognition attention bias in heroin addiction abstinence-adot-probe-based event-related potential study. Chin J Integr Med. (2011) 17:267–71.

[32] Chen YH, Ivanic B, Chuang CM, Lu DY, Lin JG. Electroacupuncture reduces cocaine-induced seizures and mortality in mice. Evid Based Complement Alternat Med. (2013) 2013:134610.

[33] Wu Jun-Mei,Wei Dong-Yan,Luo Yong-Fen,Xiang Xiao-Yong. [Clinic research on heroin de-addiction effects of acupuncture and its potentiality of preventing relapse].[J]. Zhong xi yi jie he xue bao = Journal of Chinese integrative medicine,2003,1(4):

[34] Fan AY, Miller DW, Bolash B, Bauer M, McDonald J, Faggert S, et al. Acupuncture's role in solving the opioid epidemic: evidence, cost-effffectiveness, and care availability for acupuncture as a primary, non-pharmacologic method for pain relief and management-White Paper 2017. J Integr Med. (2017) 15:411–25

[35] Wu MS, Chen KH, Chen IF, Huang SK, Tzeng PC, Yeh ML, et al. The effiffifficacy of acupuncture in postoperative pain management: a systematic review and meta-analysis. PLoS ONE. (2016) 11:e0150367.

[36] Chan YY, Lo WY, Li TC, Shen LJ, Yang SN, Chen YH, Lin JG. Clinical effiffifficacy of acupuncture as an adjunct to methadone treatment services for heroin addicts: a randomized controlled trial. Am J Chin Med. (2014) 42:569–86.

[37] Deng G, Giralt S, Chung DJ, Landau H, Siman J, Li QS, et al. Reduction of opioid use by acupuncture in patients undergoing hematopoietic stem cell

transplantation: secondary analysis of a randomized, sham-controlled trial. Pain Med. (2020) 21:636–42.

[38] Crawford P, Penzien DB, Coeytaux R. Reduction in Pain medication prescriptions and self-reported outcomes associated with acupuncture in a military patient population. Med Acupunct. (2017) 29:229–31.

[39] Baker TE, Chang G. The use of auricular acupuncture in opioid use disorder: a systematic literature review. Am J Addict. (2016) 25:592–602.

[40] Dong Y, Fan B, Yan E, et al. Decision tree model based prediction of the efficacy of acupuncture in methadone maintenance treatment. Front Neurol. 2022, Oct 6;13:956255.

[41] Wen H, Wei X, Ge S, Zeng J, Luo W, Chen R, Dong Y, Xiao S, Lai Y, Lu L. Clinical and Economic Evaluation of Acupuncture for Opioid-Dependent Patients Receiving Methadone Maintenance Treatment: The Integrative Clinical Trial and Evidence-Based Data. Front Public Health. 2021 Aug 16;9:689753.

[42] Shuqi Ge; Jiao Lan; Qu Yi; Hao Wen; Liming Lu ; Chunzhi Tang ; Acupuncture for illicit drug withdrawal syndrome: A systematic review and meta-analysis,European Journal of Integrative Medicine, 2020, 35: 101096.

[43] Wen H, Chen R, Zhang P, et al. Acupuncture for Opioid Dependence Patients Receiving Methadone Maintenance Treatment: A Network Meta-Analysis. Front Psychiatry. 2021;12:767613. Published 2021 Dec 13.

[44] American Psychiatric Association Diagnostic and Statistical Manual of Mental Disorders (DSM-V). American Psychiatric Association, Arlington, VA:2013.

[45] Wang YT, Li MC, Li KY, Xu XY, Zhuang LX. Standardized Jin's three-needle therapy for stroke: a randomized controlled trial. Zhongguo Zhen Jiu. 2023 Jan 1;43(1):9-13. Chinese.

[46] Yang X, Yu H, Zhang T, Luo X, Ding L, Chen B, Li D, Huang X, Guo X, Jia J. The effects of Jin's three-needle acupuncture therapy on EEG alpha rhythm of stroke patients. Top Stroke Rehabil. 2019 Jan 2:1-5. 10.1080/10749357.2018.1484680. Epub ahead of print. PMID: 30599806.

[47] Liao W, Tang C, Zhang J. Discussion on the principle and treatment pathway of Jin's three-needle technique for mind regulation and treatment from the "adjusting qi to regulate mind, adjusting blood to regulate mind". Zhongguo Zhen Jiu. 2018 Nov 12;38(11):1235-8. Chinese. 10.13703/j.0255-2930.2018.11.027. PMID: 30672207.

[48] Medications for Opioid Use Disorder - For Healthcare and Addiction Professionals, Policymakers, Patients, and Families (Treatment Improvement Protocol - TIP 63), https://www.ncbi.nlm.nih.gov/sites/books/NBK574910/

[49] Methadone Maintenance Treatment Program Standards and Clinical Guidelines. The college of physicians & surgeons of Ontario, Canada; 2011

[50] Wewers ME, Lowe NK. A critical review of visual analogue scales in the measurement of clinical phenomena. Res Nurs Health. 1990;13(4):227-236.

[51] Heinzerling KG, Swanson AN, Kim S, et al. Randomized, double-blind, placebo-controlled trial of modafinil for the treatment of methamphetamine dependence. Drug Alcohol Depend. 2010 Jun 1;109(1-3):20-9.

[52] Rezaei F, Emami M, Zahed S, et al. Sustained-release methylphenidate in methamphetamine dependence treatment: a double-blind and placebo-controlled trial. Daru. 2015 Jan 15;23(1):2.

[53] Canamo LJ, Tronco NB. Clinical Opioid Withdrawal Scale (COWS): Implementation and Outcomes. Crit Care Nurs Q. 2019 Jul/Sep;42(3):222-226.

[54] Buysse DJ, Reynolds CF, Monk TH, et al. The Pittsburgh Sleep Quality Index: a new instrument for psychiatric practice and research. Psychiatry Res. 1989;28(2):193-213.

[55] Tsai P-S, Wang S-Y, Wang M-Y, et al. Psychometric evaluation of the Chinese version of the Pittsburgh Sleep Quality Index (CPSQI) in primary insomnia and control subjects. Qual Life Res. 2005;14(8):1943-1952.

[56] Beck AT, Ward CH, Mendelson M, et al. Beck Depression Inventory (BDI). Arch Gen Psychiatry. 2003;4(6):504–6.

[57] Yang W-h, Liu S-l, Zhou T, Fang P. Reliability and validity of Chinese version of the Beck Depression Inventory-II in Chinese adolescents. Chin J Clin Psychol. 2014;22(2):240–5.
[58] Beck AT, Epstein N, Brown G, Steer RA. An inventory for measuring clinical anxiety: psychometric properties. J Consult Clin Psychol. 1988;56(6):893-897
[59] Thiese MS, Ronna B, Ott U. P value interpretations and considerations. J Thorac Dis. 2016 Sep;8(9):E928-E931

Study on the Clinical Efficacy and Safety of Acupuncture in Reducing Methadone Dosage in MMT clients

Informed Consent · Informed Notice page

Dear Subject:

In order to further investigate the effects of acupuncture on reducing the efficacy of methadone maintenance treatment (MMT) and improving adverse reactions to methadone, as well as provide high-quality evidence, we cordially invite your participation in the research project titled "Study on the Clinical Efficacy and Safety of Acupuncture in Reducing Methadone Dosage in MMT clients" approved by the National Natural Science Foundation in 2021.

Before you decide whether to participate in this study, kindly read the following information carefully to help you understand the purpose, procedures, duration, potential benefits, risks, and discomforts associated with this research. You may also consider discussing it with your relatives or friends or seeking explanations from your physician to aid in your decision-making process.

To safeguard the rights of participating patients, certain aspects mentioned in this document are subject to regulatory requirements and have been reviewed and approved by the ethics committee. Here is an introduction to this study:

Why is this study conducted?

Background: Opioid dependence or addiction is a grave health and societal issue, imposing a heavy burden on patients, families, and society. MMT is currently the most widely utilized therapeutic approach, requiring long treatment periods spanning several years, or even a lifetime. Methadone itself is addictive and carries the risk of abuse. Common side effects of methadone, such as insomnia, anxiety, and constipation, also afflict patients. Acupuncture, with a history of several millennia in the treatment of diseases in China, has demonstrated significant efficacy. It can regulate and calm the mind and brain, having been recommended by the World Health Organization (WHO) for the treatment of substance dependence, with notable effectiveness in treating mental disorders such as anxiety, depression, and sleep disturbances. Moreover, preliminary clinical trials conducted by the research team on 135 MMT recipients have indicated that acupuncture treatment can reduce methadone dosage, simultaneously diminishing patients' craving for the medication and averting relapse and illicit drug use caused by increased cravings resulting from dosage reduction. Additionally, it has shown positive effects in improving sleep quality and other aspects.

Objective: This study aims to further validate the clinical efficacy of acupuncture in reducing methadone dosage among MMT clients and improving adverse reactions to methadone. The goal is to provide traditional Chinese medical approaches that enable MMT recipients to reduce methadone dependence, enhance their quality of life, and facilitate their recovery by expanding the sample size.

Who should not participate in the study?

(1) complicated with serious heart, liver, spleen, lung, and kidney diseases;

(2) Patients with severe digestive diseases and severe malnutrition;

(3) patients with AIDS or syphilis;

(4) Patients with severe primary diseases of hematopoietic system;

(5) There is inflammation, scar or external injury in the operation site, or other serious systemic infected persons;

(6) Have a history of mental illness, language communication disorders;

(7) Pregnant women or those planning to become pregnant;

(8) Those who cannot cooperate or are participating in other drug trials.

How is the study conducted?

Prior to being enrolled in the study, you will be questioned by your physician, your medical history will be taken, and a physical examination will be performed to determine if you can

participate in the study. After screening, if you meet the eligibility criteria, you will be studied according to the following steps:

At the beginning of the study, the treatment you receive will be determined by random grouping based on computer software. You are 50% likely to be assigned to either the acupuncture group (experimental group) or the sham acupuncture group (control group). The sham acupuncture consists of a needle sleeve and a matching blunt acupuncture needle. The appearance of the needle is similar to that of the acupuncture group, but the difference is that the sham needle has a blunt head. Neither you nor your study physician will know which group of patients received which treatment, and neither you nor your study physician will be able to choose your treatment group. This is in order to evaluate the results more objectively. You will not be told which treatment you receive during the study.

The following are detailed descriptions of the two groups:

① Acupuncture group: acupuncture plus MMT treatment

② Control group: sham acupuncture plus MMT treatment

Acupuncture Procedure: The patient assumes a supine position, and after precise localization of the acupoints, the practitioner thoroughly disinfects their hands and the acupoints using a 75% alcohol solution. The acupuncture treatment is then administered following the prescribed protocols and guidelines for needling techniques.

Treatment course: 3 times a week, on alternate days, for 8 weeks.

During the project study, you will undergo the following checks and evaluations: Methadone effective reduction rate, clinical opioid Withdrawal Symptom Score (COWS), Visual analogue Score of Drug Craving (VAS), Pittsburgh Sleep Quality Index (PSQI), Beck Anxiety Inventory, BAI), Beck depression inventory II (BDI-II), urine morphine test.

What should I do during the research?

The study spanned a total of 31 weeks, comprising 1-week baseline assessment, an 8-week treatment phase followed by a 12-week follow-up period. Throughout this duration, you will be required to undergo various examinations and follow-up visits to the hospital in accordance with the designated schedule.

Do I have any other treatment options?

Participating in this study may or may not improve your health. You may choose to:

- Do not participate in this study, continue your regular treatment.
- Participate in other studies.
- Don't accept any treatment.

Please consult with your doctor about your decision.

How will participating in the study affect my life?

You may find this treatment inconvenient and require special arrangements. In addition, some tests may make you feel uncomfortable. If you have any questions about the tests and procedures used in the study, please consult the study physician.

During the study period, you are not allowed to receive other similar treatment methods, such as electroacupuncture, moxibustion, thread burial, etc. Consult your doctor before taking any new prescribed medications.

If you received the same type of treatment before joining the study, you must stop the treatment for at least 3 months before you can participate in our study. If you need to discontinue treatment, you should first consult your doctor to ensure your safety.

If you are a fertile woman who requires you to use contraception throughout the study period, it is recommended that you follow your doctor's advice to use reasonable contraception and avoid pregnancy. If pregnant, immediately discontinue and withdraw from the clinical study, and contact your study physician and professional obstetrician/gynecologist for appropriate treatment.

You may not participate in any other clinical studies involving drugs or medical devices during the study period.

What are the risks and adverse effects of my participation in this study?

During the acupuncture process, you may experience sensations such as soreness, tingling, heaviness, or swelling, which are normal responses to acupuncture. The most common adverse reactions associated with acupuncture treatment include mild bleeding and bruising, which typically resolve upon local compression. Additionally, dizziness caused by acupuncture may be attributed to underlying health conditions or emotional tension. In this study, all patients receive treatment in a supine position, which significantly reduces the occurrence of such discomfort. If any adverse reactions occur between visits, please consult your research physician. Furthermore, it is important to acknowledge that any treatment carries the possibility of being ineffective, and the disease may continue to progress due to treatment ineffectiveness or comorbidity with other conditions. This treatment risk is applicable to every patient, even if they are not participating in this clinical study. If the physician or researcher determines the treatment method employed in the study to be ineffective, the study will be discontinued, and an alternative potentially effective treatment method will be implemented.

You should inform your family or close friends that you are participating in a clinical study so that they can be aware of the aforementioned events. If they have any concerns about your participation in the study, you can inform them how to contact your research physician

What benefit do I get from this study?

Participating in this study may or may not improve your health.

The information gained from this study will help determine which treatment may be safer and more effective in treating other patients with conditions like yours.

What is the cost of participating in the study?

During your participation in this study, we will cover the expenses related to the study. As a token of gratitude for your inconvenience caused by participating in this research, a certain amount of remuneration will be provided to you.

Please note that the costs associated with the treatment and testing of any unrelated conditions you may have, as well as the expenses incurred by transitioning to alternative treatment methods to terminate the study, are not included. In the event of any injuries sustained during the study, we will cover the corresponding medical expenses and compensation.

What happens if I am compromised during the study?

Currently, the safety of acupuncture treatment has garnered extensive support from research data. If your health is harmed in any way related to your participation, kindly notify the research physician immediately, as they will be responsible for providing you with appropriate treatment. The research team will bear the cost of the treatment and offer corresponding financial compensation in accordance with relevant national regulations. The sponsoring party does not assume liability for medical incidents resulting from non-research-related causes or injuries caused by non-compliance with the research protocol.

Even after signing this informed consent form, you retain all your legal rights.

Is my personal information confidential?

Your medical records will be stored in the hospital, and the researchers, regulatory authorities, and ethics committee will have the authority to access your medical records. Any public reports of this study's findings will not disclose your personal identity. We will make every effort to protect the privacy of your personal medical data within the limits allowed by law.

Your personal information and medical details will be kept confidential and stored in secure locations. You may request access to your personal information, such as your name and address, at any time and make necessary modifications if required.

By signing this informed consent form, you indicate your consent to the use of your personal information and medical data for the aforementioned purposes.

Do I have to participate in the study?

This clinical trial adheres to the "Guidelines for Good Clinical Practice" and the "Helsinki Declaration," and has been approved by the Ethics Committee of the Panyu Hospital of Traditional Chinese Medicine, ensuring that your rights are protected and not infringed upon during this trial.

Participating in this study is entirely voluntary, and you have the right to refuse or withdraw from the research at any point during the study, without providing any reasons. This will not affect your relationship with your doctor, nor will it result in any loss of medical or other benefits, or subject you to any discrimination or retaliation.

Your doctor may terminate your participation in the research at any time if it is deemed necessary or in your best interest.

If you choose not to participate or withdraw from the study, there are several alternative treatment options available to you. You do not need to participate in this study to receive treatment for your condition.

If you decide to withdraw from the study for any reason, it is advisable to consult with your doctor regarding your medication usage, and you may be asked to undergo laboratory and medical examinations if deemed necessary.

If you have carefully considered and chosen to participate in this study, we hope that you will continue to complete the entire research process.

How to get more information?

(1) You may ask any questions about the study at any time during the study period.

(2) Your doctor will inform you if any important new information comes to light during the study that may affect your willingness to continue participating in the study.

What should I do now?

The decision to participate in this research is entirely yours to make. You may consider discussing it with your family or friends before deciding.

Before deciding to participate in a study, it is advisable to consult your doctor and ask as many questions as possible until you have a complete understanding of the research.

Thank you for reading the above information. If you decide to participate in this study, please inform your doctor or research assistant, who will arrange all the necessary details regarding the research on your behalf.

Please keep this information.

Informed Consent · Signature Page

Project Name: Study on the Clinical Efficacy and Safety of Acupuncture in Reducing Methadone Dosage in MMT clients

Research institute: Guangzhou University of Traditional Chinese Medicine

Trial Registration: CHiCTR2200058123

Declaration of consent

1. I have read the above introduction to this study and have had the opportunity to discuss and ask questions about this study with doctors. All my questions are answered satisfactorily.

2. I am aware of the risks and benefits that may arise from participating in this study. I understand that participation in the study is voluntary, I confirm that I have had sufficient time to consider it, and I understand that:

(1) I can always consult the doctor for more information.

(2) I can withdraw from the study at any time without discrimination or retaliation, and my medical treatment and rights will not be affected.

(3) I also know that if I were to withdraw from the study, especially if I were to withdraw due to medication, it would be in the best interest of me and the study to inform my doctor of any changes in my condition and to complete the appropriate physical and physical examination.

(4) If I need to take any other medication due to a change in my condition, I will seek my doctor's advice beforehand or tell my doctor afterwards.

(5) I consent to the drug regulatory authority, the Ethics Committee or the sponsor's representative having access to my research data.

(6) I consent □ or refuse □ Use of my medical records and pathological specimens for research other than this study.

I will obtain a signed and dated copy of the informed consent.

Finally, I decided to agree to participate in the study.

Subject's signature: _____________ Date: ___________________________

Subject's telephone number __________________________

____

I confirm that I have explained to the subject the details of the study, including their rights and possible benefits and risks, and have given them a signed copy of the informed consent.

Doctor's signature: _____________ Date: ___________________________

Doctor's telephone number: __________________________

Appendix 1: Diagnostic criteria for opioid use disorder (DSM-5)

Diagnostic Criteria for Opioid Use Disorder

A problematic pattern of opioid use leading to clinically significant impairment or distress, as manifested by at least two of the following, occurring within a 12-month period:

1. Opioids are often taken in larger amounts or over a longer period than was intended.
2. There is a persistent desire or unsuccessful efforts to cut down or control opioid use.
3. A great deal of time is spent in activities necessary to obtain the opioid, use the opioid, or recover from its effects.
4. Craving, or a strong desire or urge to use opioids.
5. Recurrent opioid use resulting in a failure to fulfill major role obligations at work, school, or home.
6. Continued opioid use despite having persistent or recurrent social or interpersonal problems caused or exacerbated by the effects of opioids.
7. Important social, occupational, or recreational activities are given up or reduced because of opioid use.
8. Recurrent opioid use in situations in which it is physically hazardous.
9. Continued opioid use despite knowledge of having a persistent or recurrent physical or psychological problem that is likely to have been caused or exacerbated by the substance.
10. Tolerance, as defined by either of the following:
    a. A need for markedly increased amounts of opioids to achieve intoxication or desired effect.
    b. A markedly diminished effect with continued use of the same amount of an opioid.
    Note: This criterion is not considered to be met for those taking opioids solely under appropriate medical supervision.
11. Withdrawal, as manifested by either of the following:
    a. The characteristic opioid withdrawal syndrome (refer to Criteria A and B of the criteria set for opioid withdrawal, pp. 547-548).
    b. Opioids (or a closely related substance) are taken to relieve or avoid withdrawal symptoms.
    Note: This criterion is not considered to be met for those individuals taking opioids solely under appropriate medical supervision.

Specify if:

In early remission:

After full criteria for opioid use disorder were previously met, none of the criteria for opioid use disorder have been met for at least 3 months but for less than 12 months (with the exception that Criterion A4, "Craving, or a strong desire or urge to use opioids," may be met).

In sustained remission:

After full criteria for opioid use disorder were previously met, none of the criteria for opioid use disorder have been met at any time during a period of 12 months or longer (with the exception that Criterion A4, "Craving, or a strong desire or urge to use opioids," may be met).

Appendix 2: General principles for dosing determination of methadone maintenance treatment

Methadone dosing in methadone maintenance treatment should be individually assessed and administered by the clinic physician based on the characteristics of the patient, taking into account factors such as the patient's physical condition, severity of drug dependence, and treatment outcome. All doses are dispensed under the direct observation of the clinic nurse and no doses are taken home.

Here are the general principles of methadone dosing in methadone maintenance treatment：

Initial Dose: The initial dose of methadone should be adequate to block cravings for other opioids and alleviate withdrawal symptoms. The starting dose typically ranges from 30 to 40 milligrams per day, depending on individual patient characteristics.

Dose Adjustment: After the initial dose, the methadone dose should be gradually adjusted based on the patient's response and needs. In general, the methadone dose is increased by 10 mg every 2 days, and as long as there are no complaints of sedation or side effects, the target dose is 60-120 mg per day.

Dose Stability: Once the patient's dose reaches a stable level that effectively controls withdrawal symptoms and cravings, clinic physician should maintain that dose while monitoring treatment efficacy and safety.

Dose reduction: methadone dose reduction should be provided when patient on dose stability stage requests a dose reduction. The rate of reduction is generally no more than 5% every two weeks. The clinic physician should closely monitor the patient's response. If adverse reactions occur, stop the methadone dose reduction process and, if necessary, return the methadone dose to the pre-tapering level.

Appendix 3: Opioid Craving VAS

Opioid craving VAS

The amount of opioid craving that a participant feels ranged across a continuum from 0 (no craving) to 100 (strong craving). The opioid craving VAS score is determined by measuring in millimeters from the left-hand end of the line to the point that the participant marks.

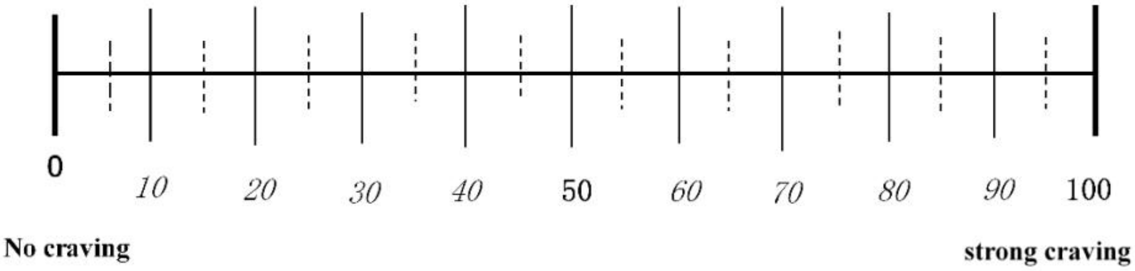

Appendix 4: Clinical Opiate Withdrawal Scale (COWS)

Clinical Opiate Withdrawal Scale (COWS)

Flow-sheet for measuring symptoms for opiate withdrawals over a period of time.

For each item, write in the number that best describes the patient's signs or symptom. Rate on just the apparent relationship to opiate withdrawal. For example, if heart rate is increased because the patient was jogging just prior to assessment, the increase pulse rate would not add to the score.

Patient's Name:____________________________ Date: ______________	
**Resting Pulse Rate**: (record beats per minute)<br>*Measured after patient is sitting or lying for one minute*<br>0 pulse rate 80 or below<br>1 pulse rate 81-100<br>2 pulse rate 101-120<br>4 pulse rate greater than 120	
**Sweating:** *over past ½ hour not accounted for by room temperature or patient activity.*<br>0 no report of chills or flushing<br>1 subjective report of chills or flushing<br>2 flushed or observable moistness on face<br>3 beads of sweat on brow or face<br>4 sweat streaming off face	
**Restlessness** *Observation during assessment*<br>0 able to sit still<br>1 reports difficulty sitting still, but is able to do so<br>3 frequent shifting or extraneous movements of legs/arms<br>5 Unable to sit still for more than a few seconds	
**Pupil size**<br>0 pupils pinned or normal size for room light<br>1 pupils possibly larger than normal for room light<br>2 pupils moderately dilated<br>5 pupils so dilated that only the rim of the iris is visible	
**Bone or Joint aches** *If patient was having pain previously, only the additional component attributed to opiates withdrawal is scored*<br>0 not present<br>1 mild diffuse discomfort<br>2 patient reports severe diffuse aching of joints/ muscles<br>4 patient is rubbing joints or muscles and is unable to sit still because of discomfort	
**Runny nose or tearing** *Not accounted for by cold symptoms or allergies*<br>*0 not present*<br>1 nasal stuffiness or unusually moist eyes<br>2 nose running or tearing<br>4 nose constantly running or tears streaming down cheeks	

COWS page 1

**GI Upset**: *over last ½ hour*<br>0 no GI symptoms<br>1 stomach cramps<br>2 nausea or loose stool<br>3 vomiting or diarrhea<br>5 Multiple episodes of diarrhea or vomiting	
**Tremor** *observation of outstretched hands*<br>0 No tremor<br>1 tremor can be felt, but not observed<br>2 slight tremor observable<br>4 gross tremor or muscle twitching	
**Yawning** *Observation during assessment*<br>0 no yawning<br>1 yawning once or twice during assessment<br>2 yawning three or more times during assessment<br>4 yawning several times/minute	
**Anxiety or Irritability**<br>0 none<br>1 patient reports increasing irritability or anxiousness<br>2 patient obviously irritable anxious<br>4 patient so irritable or anxious that participation in the assessment is difficult	
**Gooseflesh skin**<br>0 skin is smooth<br>3 piloerrection of skin can be felt or hairs standing up on arms<br>5 prominent piloerrection	
**Total scores**<br><br>**with observer's initials**	

Score:
5-12 = mild;
13-24 = moderate;
25-36 = moderately severe;
more than 36 = severe withdrawal

COWS page 2

Appendix 5: Pittsburgh Sleep Quality Index (PSQI)

PITTSBURGH SLEEP QUALITY INDEX (PSQI)

INSTRUCTIONS: The following questions relate to your usual sleep habits during the past month only. Your answers should indicate the most accurate reply for the majority of days and nights in the past month. Please answer all questions.

entry	project	score			
		0	1	2	3
1	During the past month, when have you usually gone to bed at night?	**USUAL BED TIME** ______			
2	During the past month, when have you usually gotten up in the morning?	**USUAL GETTING UP TIME** ______			
3	During the past month, how many hours of actual sleep did you get at night? (This may be different than the number of hours you spend in bed.)	**USUAL GETTING UP TIME** ______			
4	During the past month, how long (in minutes)has it usually take you to fall asleep each night?	☐≤15minutes	☐16-30minutes	☐31-60minutes	☐≥60minutes
5	INSTRUCTIONS: For each of the remaining questions, check the one best response.Please answer all questions.				
	During the past month, how often have you had trouble sleeping because you...	Not during the past month	Less than once a week	Once once or twice a week	Three or more times a week
	(a)...cannot get to sleep within 30 minutes	☐	☐	☐	☐
	(b)...wake up in the middle of the night or early morning	☐	☐	☐	☐
	(c)...have to get up to use the bathroom	☐	☐	☐	☐
	(d ...cannot breathe comfortably	☐	☐	☐	☐
	(e)...cough or snore loudly	☐	☐	☐	☐
	(f)...feel too cold	☐	☐	☐	☐
	(g)...feel too hot	☐	☐	☐	☐
	(h)...had bad dreams	☐	☐	☐	☐
	(i)...have pain	☐	☐	☐	☐
	(j)Other reason(s),	☐	☐	☐	☐
	please describe:				
6	During the past month,how would you	☐ Very good	☐Fairly good	☐ Fairly bad	☐ Very bad

PSQI page 1

	rate your sleep quality overall?				
		Less than once a week	Once once or twice a week	Three or more times a week	Less than once a week
7	During the past month,how often have you taken medicine(prescribed or "over the counter")to help you sleep?	☐	☐	☐	☐
8	During the past month,how often have you had trouble staying awake while driving. eating meals,or engaging in social activity?	☐	☐	☐	☐
9	During the past month, how much of a problem has it been for you to keep up enough enthusiasm to get things done?	☐ No problem at all	☐ Only a very slight problem	☐ Somewhat of a problem	☐ A very big problem

Appendix 6: The Beck Anxiety Inventory (BAI)

Beck Anxiety Inventory (BAI)

Below is a list of common symptoms of anxiety. Please carefully read each item in the list. Indicate how much you have been bothered by that symptom during the past month, including today, by circling the number in the corresponding space in the column next to each symptom.

	Not at all	Mildly, but it didn't bother me much	Moderately – it wasn't pleasant at times	Severely – it bothered me a lot
Numbness or tingling	0	1	2	3
Feeling hot	0	1	2	3
Wobbliness in legs	0	1	2	3
Unable to relax	0	1	2	3
Fear of worst happening	0	1	2	3
Dizzy or lightheaded	0	1	2	3
Heart pounding / racing	0	1	2	3
Unsteady	0	1	2	3
Terrified or afraid	0	1	2	3
Nervous	0	1	2	3
Feeling of choking	0	1	2	3
Hands trembling	0	1	2	3
Shaky / unsteady	0	1	2	3
Fear of losing control	0	1	2	3
Difficulty in breathing	0	1	2	3
Fear of dying	0	1	2	3
Scared	0	1	2	3
Indigestion	0	1	2	3
Faint / lightheaded	0	1	2	3
Face flushed	0	1	2	3
Hot / cold sweats	0	1	2	3

Appendix 7: Beck Depression Inventory-II (BDI-II)

BDI-II

Instructions: This questionnaire consists of 21 groups of statements. Please read each group of statements carefully. And then pick out the one statement in each group that best describes the way you have been feeling during the past two weeks, including today. Circle the number beside the statement you have picked. If several statements in the group seem to apply equally well, circle the highest number for that group. Be sure that you do not choose more than one statement for any group, including Item 16 (Changes in Sleeping Pattern) or Item 18 (Changes in Appetite).

1. Sadness
   0. I do not feel sad.
   1.I feel sad much of the time.
   2.I am sad all the time.
   3.I am so sad or unhappy that I can't stand it.

2. Pessimism
   0. I am not discouraged about my future.
   1.I feel more discouraged about my future than I used to.
   2.I do not expect things to work out for me.
   3.I feel my future is hopeless and will only get worse.

3.Past Failure
   0. I do not feel like a failure.
   1.I have failed more than I should have.
   2.As I look back, I see a lot of failures.
   3.I feel I am a total failure as a person.

4. Loss of Pleasure
   0. I get as much pleasure as I ever did from the things I enjoy.
   1. I don't enjoy things as much as I used to.
   2. I get very little pleasure from the things I used to enjoy.
   3. I can't get any pleasure from the things I used to enjoy.

5.Guilty Feelings
   0. I don't feel particularly guilty.
   1. I feel guilty over many things I have done or should have done.
   2. I feel quite guilty most of the time.
   3.I feel guilty all of the time.

BDI- II page 1

6.Punishment Feelings

0. I don't feel I am being punished.
1. I feel I may be punished.
2. I expect to be punished.
3. I feel I am being punished.

7.Self-Dislike

0. I feel the same about myself as ever.
1. I have lost confidence in myself.
2. I am disappointed in myself.
3. I dislike myself.

8.Self-Criticalness

0. I don’t criticize or blame myself more than usual.
1. I am more critical of myself than I used to be.
2. I criticize myself for all of my faults.
3. I blame myself for everything bad that happens.

9.Suicidal Thoughts or Wishes

0. I don*t have any thoughts of killing myself.
1. I have thoughts of killing myself, but I would not carry them out.
2. I would like to kill myself.
3. I would kill myself if I had the chance.

10.Crying

0. I don't cry anymore than I used to.
1. I cry more than I used to.
2. I cry over every little thing.
3. I feel like crying, but I can't.

11. Agitation

0. I am no more restless or wound up than usual.
1. I feel more restless or wound up than usual.
2. I am so restless or agitated, it's hard to stay still.
3. I am so restless or agitated that I have to keep moving or doing something.

12. Loss of Interest

0. I have not lost interest in other people or activities.
1. I am less interested in other people or things than before.
2. I have lost most of my interest in other people or things.
3. It's hard to get interested in anything.

13. Indecisiveness

0. I make decisions about as well as ever.
1. I find it more diffiicult to make decisions than usual.
2. I have much greater difficulty in making decisions than I used to.
3. I have trouble making any decisions.

14.Worthlessness

0. I do not feel I am worthless.
1. I don't consider myself as worthwhile and useful as I used to.
2. I feel more worthless as compared to others.
3. I feel utterly worthless.

15. Loss of Energy

0. I have as much energy as ever.
1. I have less energy than I used to have.
2. I don't have enough energy to do very much.
3. I don't have enough energy to do anything.

16. Changes in Sleeping Pattern

0. I have not experienced any change in my sleeping. 1a I sleep somewhat more than usual.
1. I sleep somewhat less than usual,or I sleep somewhat greater than usual.
2. I sleep a lot more than usual,or I sleep a lot less than usual.
3. I sleep most of the day,or I wake up 1-2 hours early and can't get back to sleep.

17.Irritability

0. I am not more irritable than usual.
1. I am more irritable than usual.
2. I am much more irritable than usual.
3. I am irritable all the time.

18.Changes in Appetite

0. I have not experienced any change in my appetite.
1. My appetite is somewhat less than usual,or my appetite is somewhat greater than usual.
2. My appetite is much less than before,or my appetite is much greater than usual.
3. I have no appetite at all ,or I crave food all the time.

19.Concentration Difficulty

0. I can concentrate as well as ever.
1. I can't concentrate as well as usual.

2. It's hard to keep my mind on anything for very long.
3. I find I can't concentrate on anything.

20.Tiredness or Fatigue

0. I am no more tired or fatigued than usual.
1. I get more tired or fatigued more easily than usual.
2. I am too tired or fatigued to do a lot of the things I used to do.
3. I am too tired or fatigued to do most of the things I used to do.

21.Loss of Interest in Sex

0. I have not noticed any recent change in my interest in sex.
1. I am less interested in sex than I used to be.
2. I am much less interested in sex now.
3. I have lost interest in sex completely.

Total Score:

Statistical Analysis Plan

1. Introduction

Opioid Use Disorder (OUD) represents an addictive condition characterized by intense cravings for opiate substances, increased tolerance, difficulty in reducing usage, and notable withdrawal symptoms upon cessation. According to the "World Drug Report 2021", as of 2020, approximately 275 million individuals globally were drug users, with opiate drugs still contributing the most to the disease burden caused by illicit substances.

Methadone, an artificially synthesized opioid medication, is the cornerstone of methadone maintenance treatment (MMT), a community-based substitution therapy for opioid substances. The effectiveness of MMT in treating OUD is unquestionable, but associated issues have become increasingly apparent. As a synthetic opioid medication, methadone carries addictive potential and the risk of misuse. As the duration of treatment progresses, tolerance to methadone also increases, necessitating higher doses to maintain therapeutic effects, thereby raising the risk of overdose-related fatalities. Moreover, reports indicate a persistent rise in constipation among MMT clients, with insomnia detection rates ranging from 58.2% to 85.8%. Patients experience varying degrees of anxiety, depression, and other symptoms for a considerable period, while MMT-related sexual dysfunction is also prevalent. MMT clients have a need to reduce the methadone dose, but the effectiveness of reducing the methadone dose by themselves is limited. Currently, there is still a lack of measures to promote the reduction of methadone dosage while reducing its risk of opioid relapse.

Acupuncture, as an integral component of traditional Chinese medicine, boasts a history spanning thousands of years in disease treatment. Several studies have identified the potential role of acupuncture in improving opioid dependence by reducing opioid use and diminishing craving. In general, although there have been several studies focusing on acupuncture as an adjunct treatment for drug addiction,

they have predominantly consisted of small-sample clinical trials without the inclusion of sham acupuncture as a control. Consequently, there is a compelling need to undertake high-quality prospective RCT incorporating sham acupuncture as a control in order to substantiate and affirm the efficacy and safety of acupuncture in MMT, thereby providing more persuasive evidence.

2. Study Objectives and Hypothesis

The aim of this study is to evaluate the efficacy and safety of manual acupuncture intervention for MMT clients through a multicenter clinical randomized controlled trial, comparing it with non-penetrating sham acupuncture.

3. Design

3.1 Overview

This parallel-arm, patient-blinded, multicenter, randomized, controlled trial will be performed to demonstrate the safety and effectiveness of manual acupuncture intervention for MMT clients.

3.2 Eligibility Criteria

3.2.1 Inclusion Criteria

Patients will be included if they have:

- Diagnosed as opioid dependence according to the fifth edition of the Diagnostic and Statistical Manual of Mental Disorders (DSM-5) (Appendix 1);
- Aged between18-65 years;
- Received MMT for more than 6 weeks;
- Demand for methadone tapering;
- Not having received any kind of acupuncture therapy during the previous 3 months.

3.2.2 Exclusion Criteria

Patients will be excluded if they have:

- Serious heart, liver, lung, or kidney diseases;
- Syphilis or AIDS;
- Severe digestive disease or malnutrition;
- Severe primary hematological disorders;
- Received other treatment that may affect the efficacy evaluation of the present intervention;
- Infection, inflammation, scar, or injury close to the site of the selected acupoints;
- Pregnancy, or plans for pregnancy;
- Had a history of mental illness other than drug dependence.

In addition, all patients are instructed not to take any other medications aiming at alleviating withdrawal symptoms, improving sleep quality, or managing depression and anxiety during the study, in order to avoid initiating other interventions. Patients who consume such medications are excluded from the study.

4. Study Schema

	STUDY PERIOD												
	Enrolment	Allocation	Treatment phase								Follow-up		
**Timepoint (week)**	−1	0	1	2	3	4	5	6	7	8	12	16	20
**ENROLLMENT:**	×												
Eligibility screen	×												
Informed consent	×												
Allocation		×											
**INTERVENTIONS:**													
Manual Acupuncture			◆	———	———	———	———	———	———	▶			
Sham Acupuncture			◆	———	———	———	———	———	———	▶			
**ASSESSMENTS:**													
Daily Consumption of Methadone		×		×		×		×		×	×	×	×
opioid craving VAS		×		×		×		×		×	×	×	×
COWS		×		×		×		×		×	×	×	×
BAI		×				×				×	×	×	×
BDI-II		×				×				×	×	×	×
PSQI		×				×				×	×	×	×
**ADVERSE EVENTS**:			×	×	×	×	×	×	×	×	×	×	×

Abbreviations: Opioid craving VAS, the visual analog scale for opioid craving; COWS, Clinical Opioid Withdrawal Scale; PSQI, Pittsburgh Sleep Quality Index; BDI-II, The Beck Depression Inventory-II; BAI, The Beck Anxiety Inventory.

Figure 1: Schedule of enrolment, interventions, and assessments

5. Efficacy and Safety Outcomes

5.1 Primary Outcomes

One primary outcome is the rate of methadone dose reduction, defined as the proportion of participants who achieve a reduction in methadone dose of ≥20% compared to baseline after 8 weeks of intervention. The other primary outcome is the opioid craving using a visual analog scale for drug craving marking on a 100-millimetre line in proportion to their craving from 0 (no craving) to 100(strong craving)

5.2 Secondary Outcomes

- Clinical Opioid Withdrawal Scale (COWS)
- Pittsburgh sleep quality index (PSQI)
- The beck depression inventory-II (BDI-II)
- The beck anxiety inventory (BAI)
- Urine test

6. Statistical Considerations

6.1 Study Hypothesis

The primary study hypothesis is that manual acupuncture is more efficacy than sham acupuncture in reducing methadone dose and opioids craving in MMT clients.

6.2 Study Populations

All randomised patients who receive at least one session of acupuncture intervention and at least one efficacy measure will be included in the full analysis set. All randomised patients who completed all the intervention and 20 weeks follow-up assessments will be included in per-protocol analysis set.

7. Statistical Analysis

7.1 Summary Statistics

Continuous variables are reported as the mean with standard deviation or median and interquartile range. Categorical variables are described as numbers and percentages. Continuous variables will be compared using a two-sample t-test or Wilcoxon rank-sum test if data show serious deviations from a normal distribution. Categorical data or ordinal data will be compared using a Wilcoxon rank-sum test, chi-square test or Fisher's exact test, as appropriate.

7.2 Missing Data

We will use multiple imputation method under the missing at random (MAR) assumption for the primary and secondary outcome with missing data. Multiple imputations use the observed data to fill in the missing values repeatedly to give rise to multiple "pseudo-complete" datasets. We will perform the imputation of missing data 100 times using regression imputation, employing the SAS procedure Proc MI process. For each time point, measurements of primary and secondary outcomes are imputed using groups, baseline measurements, and measurements prior to the time point as covariates in regression models. The estimated values are set as integers, and 100 different datasets are generated. By calculating the median of all estimated values for missing data, the 100 imputed datasets are merged into one.

7.3 Demographics and Baseline Characteristics

All data recorded at baseline will be summarized by group. Comparisons between groups will be done using the methodology described in section 7.1.

7.4 Analyses for Outcomes

The analysis of proportion of participants with at least 20% reduction from baseline in the amount of methadone dose is performed using a generalized linear mixed model. For opioid craving VAS and all secondary outcomes, analysis is conducted utilizing a

linear mixed-model. For above models, values measured at baseline are used as covariates; intervention assignment, visit and intervention × visit interaction are as fixed effects; and treatment centre and centre × intervention interaction are as random effects. Before building the final model, we assess the poolability of treatment effects across centres by including centre × intervention interaction in the model. If *P*-values corresponding to the interaction term for both primary endpoints are greater than 0.10, the treatment effect is homogenous across centres and centre × intervention interaction will not be included in the final model. Primary outcomes use 2-sided tests at the 2.5% significance level. All other statistical tests are 2-sided at the 5% significance level. Dynamic data on the reduction in the methadone consumption and the opioid craving VAS score will be demonstrated in the figures, separated by two groups. Each row in the figures represents the reduction dose of the daily consumption of methadone (the opioid craving VAS score) for one participant. To investigate the relationship between daily consumption of methadone and the intensity of opioid craving over a 20-week period, scatter plots will be employed as a visual representation in this study with the x-axis representing the opioid craving VAS and the y-axis representing the methadone dose. The resulting scatter plot allows for the examination of the correlation between these two variables, providing insights into the potential influence of methadone intake on opioid craving levels over time.

7.5 Sensitivity Analyses for the Primary Outcomes

A PP analysis is carried out as a sensitivity analysis compared with the results obtained using the FAS analysis. When the PPs and FAS analysis conclusions are not the same, it is necessary to analyze the possible reasons and discussion them.

7.6 Safety Analyses

All adverse events and serious adverse events will be listed. Adverse events include the acupuncture-related adverse events and other adverse events. Chi-square test or Fisher’s exact test will be used to compare the incidence of adverse events between the manual acupuncture and sham control groups.

7.7 Blinding Assessment

The number and percentage of subjects who answer what kind of intervention they feel they have received (Acupuncture/Sham/Uncertain) will be summarized. Chi-square test or Fisher's exact test will be used to compare the difference of blinding assessment between the two groups.

7.8 Analysis Software

Statistical analyses are conducted by an independent statistician using SAS version 9.4 (SAS Institute Inc, Cary, NC), including SAS PROC GLIMMIX for generalized linear mixed model, and SAS PROC MIXED for linear mixed-model.
